# PD‐1^hi^CXCR5^−^CD4^+^T Peripheral Helper Cells Were Enriched and Potentially Predicted Clinical Response to Etanercept Therapy in Rheumatoid Arthritis

**DOI:** 10.1002/mco2.70876

**Published:** 2026-07-30

**Authors:** Haojie Xu, Wanki Ho, Lulu Cao, Dongdong Fu, Yun Li, Feng Sun, Xi Xu, Zhanguo Li, Ru Li, Hua Ye, Huaqun Zhu

**Affiliations:** ^1^ Department of Rheumatology and Immunology Peking University People's Hospital Beijing China; ^2^ Beijing Key Laboratory of Non‐invasive Diagnosis and Immunotherapy of Rheumatic Diseases Beijing China

**Keywords:** disease activity, efficacy prediction, rheumatoid arthritis, TNF‐α inhibitor, T peripheral helper cell

## Abstract

Rheumatoid arthritis (RA) remains a challenging autoimmune disease with variable treatment responses to tumor necrosis factor‐α (TNF‐α) inhibitors. This study investigates the clinical significance of PD‐1^hi^CXCR5^−^CD4^+^T peripheral helper (Tph) cells in RA and their potential utility as biomarkers for predicting etanercept (ETN) therapy response. We enrolled 58 RA patients, 12 age‐ and sex‐matched osteoarthritis patients, and 15 healthy controls, with Tph cells quantified by flow cytometry. Among 25 RA patients with inadequate response to conventional synthetic disease‐modifying antirheumatic drugs receiving ETN therapy, treatment outcomes were stratified by ACR20 response criteria. Tph cell frequency was significantly elevated in RA patients and correlated positively with multiple disease activity indicators. At baseline, ETN nonresponders exhibited higher Tph proportions than responders (13.23 ± 2.60% vs. 10.82 ± 3.08%, *p* = 0.0467). Following ETN treatment, responders demonstrated significant Tph reduction (10.82% decreased to 7.97%, *p* = 0.0105), paralleling serum IL‑21 dynamics. In an exploratory analysis, baseline Tph levels showed an association with ETN response. These findings suggest that circulating Tph cells may serve as a candidate biomarker for RA disease activity and therapeutic response monitoring, warranting further validation in larger prospective cohorts.

## Graphical abstract

This study investigated the role of PD‐1^hi^CXCR5^−^CD4^+^ T peripheral helper (Tph) cells in rheumatoid arthritis (RA). Tph cells were increased in the peripheral blood of RA patients and associated with disease activity. Baseline Tph cell levels were linked to the response to etanercept therapy. In responders, Tph cell frequencies and serum IL‐21 levels decreased after treatment. Tph cells may serve as a potential biomarker for monitoring RA disease activity and therapeutic response.

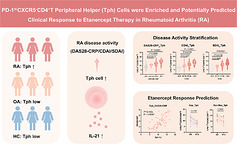

## 1 Introduction

Rheumatoid arthritis (RA) is a chronic, systemic, autoimmune inflammatory disease primarily affecting the joints and periarticular soft tissues [1]. It is characterized by persistent synovial hyperplasia, inflammatory cell infiltration, and pannus formation, which commonly leads to symmetrical joint pain, swelling, morning stiffness, and progressive functional impairment [2]. Without timely and standardized intervention, recurrent inflammation will eventually result in irreversible joint destruction, deformity, and even permanent disability, bringing a heavy physical, psychological, and economic burden to patients and society [3, 4]. Tumor necrosis factor‐α inhibitors (TNF‐αi) have become the mainstream biological agents for RA treatment since their clinical application, and have greatly improved the long‐term prognosis of patients by effectively blocking inflammatory cascades and alleviating synovitis [5]. Nevertheless, clinical practice confirms that approximately 20%–40% of RA patients fail to achieve satisfactory therapeutic responses after receiving TNF‐αi therapy [6]. At present, there is still a lack of reliable, clinically validated biomarkers to accurately predict individual treatment responses in RA patients, which hinders the implementation of individualized treatment strategies for RA [7]. Therefore, in‐depth exploration of the pathological mechanism of RA and identification of novel, accessible biomarkers are of great clinical significance for evaluating disease status and treatment responses, thereby promoting the implementation of personalized therapy for RA.

Aberrant activation of adaptive immunity remains central to RA pathogenesis [8]. Early studies are predominantly focused on the pathogenic roles of T helper 1 (Th1 cells) and T helper 17 (Th17) cells in joint inflammation, and clarified their regulatory effects on pro‐inflammatory cytokine secretion and immune cell recruitment [9, 10]. However, with the rapid development of high‐resolution molecular biology technologies, a variety of previously unrecognized CD4^+^ T cell subsets in synovial tissues and peripheral blood have been gradually discovered [11, 12, 13], and these newly identified immune populations are proven to participate in the whole process of RA inflammation. Peripheral T helper (Tph) cells, a novel CD4^+^T cell subset defined by immunophenotype PD‐1^hi^CXCR5^−^CD4^+^, are mainly enriched in inflamed joint tissues of RA patients [14]. These cells predominantly produce interleukin‐21 (IL‐21), a pivotal cytokine that promotes B‐cell differentiation, antibody production, and germinal center formation [15]. Existing studies have confirmed that Tph cells can promote the construction of tertiary lymphoid structures in local lesions, facilitate autoantigen‐specific B‐cell differentiation, and continuously amplify autoimmune inflammatory responses [15, 16, 17]. Given its unique immunophenotype and biological function, Tph cells have gradually become an intriguing candidate for exploring the immune mechanism of RA.

Emerging evidence suggests that Tph cells may hold clinical utility in several aspects of RA management [14]. Recent studies have reported associations between Tph cell frequencies and disease activity measures, and preliminary data hint at their potential role in predicting progression from preclinical autoimmunity to clinical RA [18]. Several exploratory reports remain insufficient to establish Tph cells as independent predictors of RA disease activity or treatment response [19]. These gaps highlight the necessity for more rigorous, hypothesis‐testing research to establish Tph cells as clinically actionable biomarkers.

Against this backdrop, the present study mainly aims to explore the clinical significance of circulating Tph cells in RA patients and preliminarily verify their potential value in predicting therapeutic responses to etanercept (ETN, a classic TNF‐αi) [20]. As an exploratory finding in the present study, our data suggested an independent link between Tph cells and RA disease activity, and implied that circulating Tph cells may have the potential to reflect ETN treatment response. Further large‐sample prospective studies are still warranted to validate our observations and support the clinical application of Tph cells.

## 2 Results

### 2.1 Tph Cells Were Significantly Enriched in the Peripheral Blood of RA Patients

The demographic and clinical characteristics of the RA patients at baseline are summarized in Table S1. Baseline age and sex distribution among RA, osteoarthritis (OA), and healthy controls (HC) groups were comparable (Table S2). Among the 58 RA patients, disease activity states were distributed as follows: 20 were in complete remission (DAS28‐CRP < 2.6), eight had low disease activity (2.6 ≤ DAS28‐CRP ≤ 3.2), 18 had moderate disease activity (3.2 < DAS28‐CRP ≤ 5.1), and 12 had high disease activity (DAS28‐CRP > 5.1).

**FIGURE 1:**
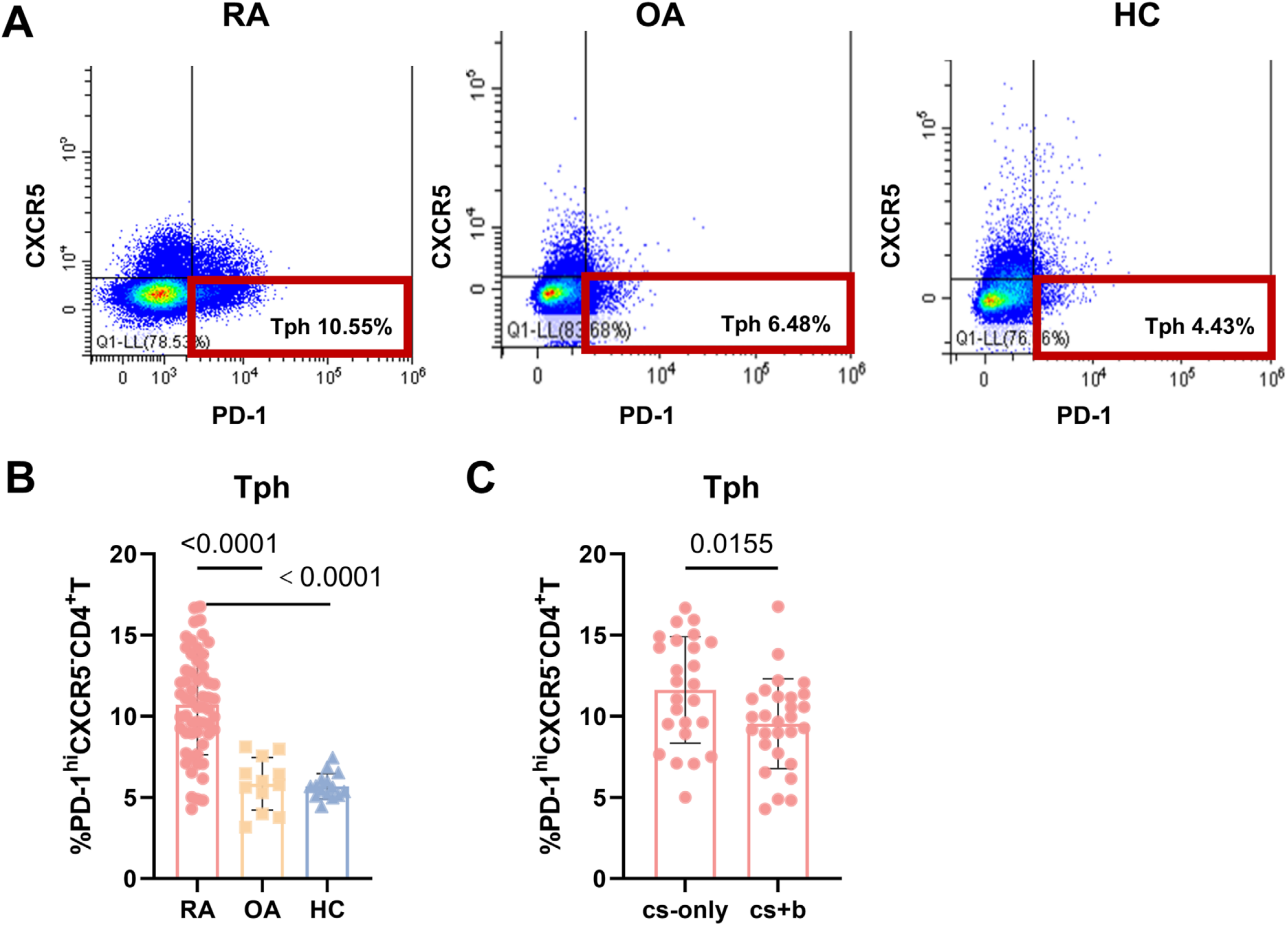
(A) Flow cytometry analysis revealed markedly enriched Tph cells in the peripheral blood of RA patients compared to those of OA patients and HC. (B) The percentage of Tph cells was significantly higher in the RA group than in the OA or HC group. (C) The percentage of Tph cells was significantly higher in the csDMARDs‐only (cs‐only) treatment group than in the csDMARDs plus biologic DMARDs (bDMARDs, cs+b) combination therapy group. bDMARDs, biologic disease‐modifying antirheumatic drugs; csDMARDs, conventional synthetic disease‐modifying antirheumatic drugs; HC, healthy controls; OA, osteoarthritis; RA, rheumatoid arthritis; Tph cells, PD‐1^hi^CXCR5^−^CD4^+^T peripheral helper cells.

Flow cytometry analysis revealed a marked enrichment of Tph cells in the peripheral blood of RA patients compared to OA patients and HC (Figure 1A). The frequency of Tph cells among peripheral blood CD4^+^T cells was significantly higher in RA patients than in OA patients or HC (10.73 ± 3.11% vs. 5.85 ± 1.62% vs. 5.69 ± 0.78%, *p* < 0.0001) (Figure 1B). Furthermore, RA patients were stratified into four groups based on prior treatment: an untreated group (*n* = 3), a conventional synthetic disease‐modifying anti‐rheumatic drugs (csDMARDs)‐only group (*n* = 25), a combination therapy group receiving csDMARDs plus biologic DMARDs (bDMARDs) (*n* = 28), and a group receiving csDMARDs plus targeted synthetic DMARDs (tsDMARDs) (*n* = 2). Given the very small sample sizes, formal statistical comparisons were not performed for the untreated (*n* = 3) and csDMARDs+tsDMARDs (*n* = 2) subgroups.​ The frequency of Tph cells was significantly higher in the csDMARDs‐only group than in the csDMARDs plus bDMARDs combination therapy group (11.63 ± 3.29% vs. 9.54 ± 2.76%, *p* = 0.0155, Figure 1C). Additional stratification by csDMARD type (MTX monotherapy, other csDMARDs monotherapy, MTX+other csDMARDs) was performed, showing no significant Tph differences across subgroups (Figure S1).

We further analyzed the correlation between Tph cell frequency and clinical/laboratory parameters. Tph cell percentage showed a positive correlation with white blood cells (WBC) (*r* ^2^ = 0.086, *p* = 0.0273) and platelets (PLT) (*r* ^2^ = 0.106, *p* = 0.0135), and a negative correlation with hemoglobin (HGB) (*r* ^2^ = 0.068, *p* = 0.0495) (Figure S2A–C). No significant correlations were found between Tph cell frequency and other clinical data (age, sex, disease duration, smoking history, and alcohol consumption) (Figure S3A–E), or other laboratory parameters (alanine transaminase [ALT], blood urea nitrogen [BUN], creatinine [Cr], rheumatoid factor [RF], and anti‐cyclic citrullinated peptide antibody [Anti‐CCP Ab]) (Figure S3F–J).

### 2.2 Tph Cells Correlated With RA Disease Activity

**FIGURE 2:**
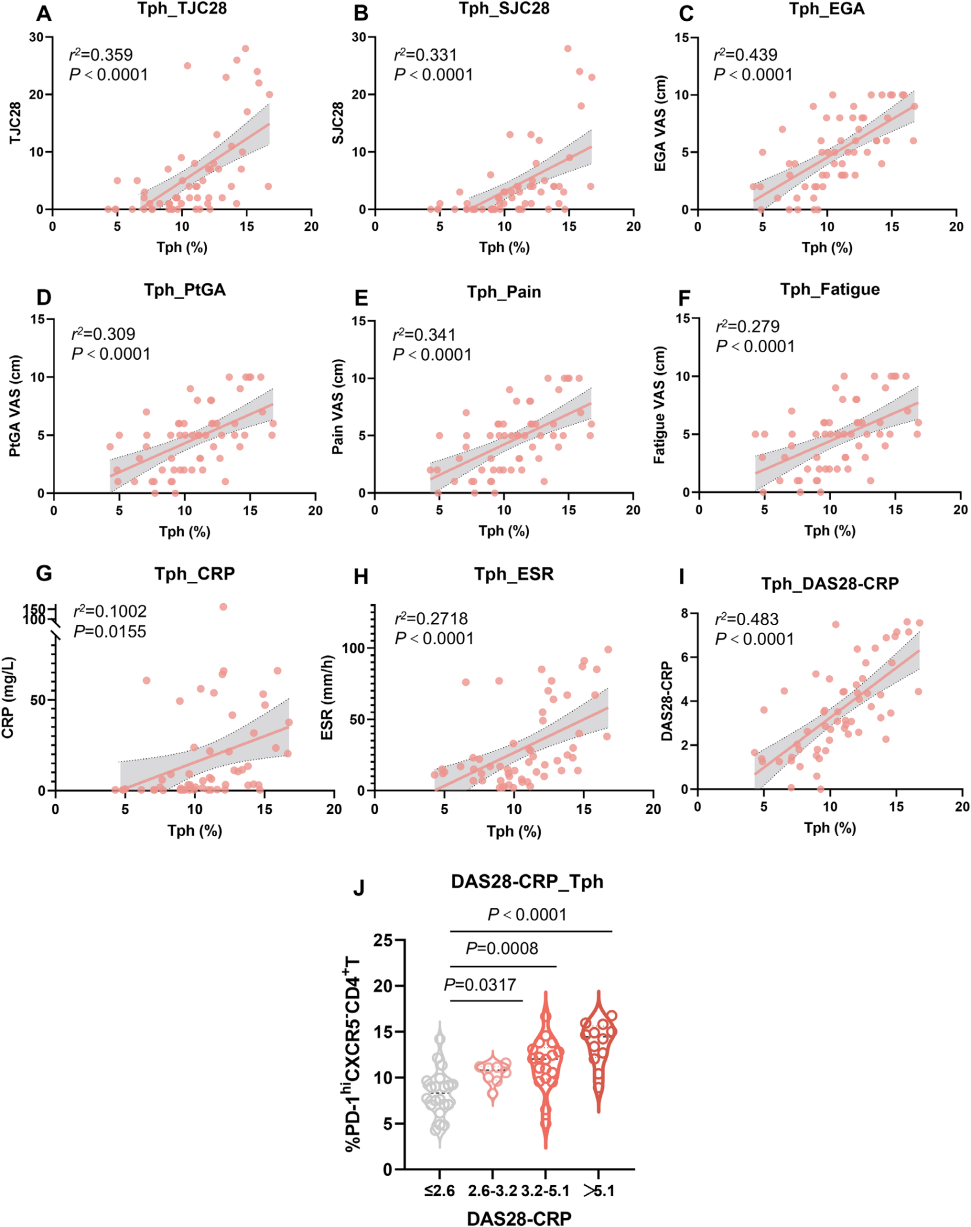
(A–F) The percentage of Tph cells was positively correlated with TJC28, SJC28, EGA VAS, PtGA VAS, Pain VAS. and Fatigue VAS. (G, H) The percentage of Tph cells was positively correlated with CRP and ESR, respectively. (I) The percentage of Tph cells was positively correlated with DAS28‐CRP. (J) The percentage of Tph cells progressively increased with higher DAS28‐CRP. DAS28‐CRP, 28‐joint disease activity score based on C‐reactive protein; EGA VAS, evaluator global assessment visual analog scale; PtGA VAS, patient global assessment visual analog scale; SJC28, 28‐joint swollen joint count; TJC28, 28‐joint tender joint count; VAS, visual analog scale.

Our analysis revealed that the frequency of Tph cells showed positive correlations with multiple clinical measures of RA disease activity, including TJC28 (*r* ^2^ = 0.359, *p* < 0.0001), SJC28 (*r* ^2^ = 0.331, *p* < 0.0001), EGA VAS (*r* ^2^ = 0.439, *p* < 0.0001), PtGA VAS (*r* ^2^ = 0.309, *p* < 0.0001), Pain VAS (*r* ^2^ = 0.341, *p* < 0.0001), and Fatigue VAS (*r* ^2^ = 0.279, *p* < 0.0001) (Figure 2A–F). Tph cell frequency also demonstrated strong positive correlations with established inflammatory markers: C‐reactive protein (CRP) (*r* ^2^ = 0.1002, *p* = 0.0155) and erythrocyte sedimentation rate (ESR) (*r* ^2^ = 0.2718, *p* < 0.0001) (Figure 2G,H).

Given these robust correlations with disease activity indicators, we further examined the relationship between Tph cells and composite disease activity assessment tools (28‐Joint Disease Activity Score based on C‐reactive protein, DAS28‐CRP, *r* ^2^ = 0.483, *p* < 0.0001) (Figure 2I). Tph cell levels showed a progressive increase across the four‐category DAS28‐CRP classification (remission/low/moderate/high activity) (Figure 2J). Tph cell frequency demonstrated comparable positive correlations with additional composite disease activity indices: Clinical Disease Activity Index (CDAI) (*r* ^2^ = 0.439, *p* < 0.0001) and Simplified Disease Activity Index (SDAI) (*r* ^2^ = 0.264, *p* < 0.0001) (Figure S4). Tph cells similarly showed progressive increases across the four‐category classifications of both CDAI and SDAI, paralleling the trend observed with DAS28‐CRP.

### 2.3 Tph Cells Were Independently Associated With Active RA

Having established that Tph cell frequency correlates with multiple measures of RA disease activity, we next sought to determine whether Tph cells were independently associated with disease activity status, even when accounting for other immune cell subsets. For this purpose, patients were categorized into remission (DAS28‐CRP < 2.6, *n* = 20) and active disease (DAS28‐CRP ≥ 2.6, *n* = 38). Univariate binary logistic regression analysis was performed with disease activity status as the dependent variable, and Tph cell percentage along with other immune cell subsets as independent variables. Multivariate binary logistic regression analysis was further conducted to identify independent factors (Table 1, Table S3).

**TABLE 1:** Univariate and multivariate binary logistic regression analyses for the association of Tph cells and other immune cell subsets with DAS28‐CRP‐defined disease activity in RA.

Variable	Univariate analysis OR (95% CI)	*p‐*value	Multivariate analysis OR (95% CI)	*p‐*value
Tph (%)	1.72 (1.28–2.32)	<0.001	2.12 (1.03–4.37)	0.040
TNF‐α^+^Th (%)	0.94 (0.88–1.00)	0.058	1.00 (0.87–1.15)	0.976
IFN‐γ^+^Th (%)	0.92 (0.85–1.01)	0.067	1.04 (0.85–1.28)	0.673
IL‐2^+^Th (%)	0.99 (0.93–1.05)	0.773	0.99 (0.82–1.19)	0.904
Th17 (%)	0.62 (0.22–1.78)	0.377	0.15 (0.02–1.45)	0.103
CD161^+^Treg (%)	0.97 (0.88–1.07)	0.513	0.98 (0.81–1.18)	0.818
Foxp3^+^ (%)	1.09 (0.74–1.61)	0.661	0.63 (0.16–2.47)	0.512
Teff (%)	0.93 (0.71–1.22)	0.602	1.20 (0.57–2.53)	0.637
Naïve Th (%)	1.01 (0.96–1.06)	0.683	0.93 (0.81–1.08)	0.344
Tfh (%)	1.28 (0.90–1.82)	0.175	1.29 (0.57–2.91)	0.546

Abbreviations: CD161, cluster of differentiation 161; CI, confidence interval; Foxp3, forkhead box P3; IFN‐γ, interferon‐γ; IL‐2, interleukin‐2; Naïve Th, naive T helper cells; OR, odds ratio; RA, rheumatoid arthritis; Teff, effector T cells; Tfh, T follicular helper cells; Th17, T helper 17 cells; TNF‐α, tumor necrosis factor‐α; Tph, T peripheral helper cells; Treg, regulatory T cells.

Univariate analysis showed that Tph cell percentage was significantly associated with DAS28‐CRP‐defined disease activity. In the subsequent multivariate model including Tph cell percentage and other immune subsets, only Tph cell percentage remained independently associated with disease activity, suggesting that Tph cell percentage may be an independent factor associated with RA disease activity in this exploratory model, a finding that requires validation. To evaluate the diagnostic performance of Tph cells in distinguishing disease activity status, receiver operating characteristic (ROC) curves were constructed. The area under the curve (AUC) for Tph cells was 0.85 (95% CI: 0.74–0.96) (Figure S5). For exploratory reference only, a candidate Tph cell cut‐off value for differentiating active from remission RA was 9.41% (sensitivity 90%, specificity 75%), which requires prospective validation in larger cohorts.

### 2.4 Tph Cells Were Associated With Clinical Response to ETN Therapy in an Exploratory Analysis

Next, we followed 25 RA patients receiving ETN therapy. Based on ACR20 response at Week 12, patients were categorized into ETN responders (*n* = 13) and nonresponders (*n* = 12). At baseline, nonresponders exhibited a higher frequency of Tph cells and Tfh cells than responders (Tph: 13.23 ± 2.60% vs. 10.82 ± 3.08%, *p* = 0.0467; Tfh: 4.04 ± 2.14% vs. 2.44 ± 0.95%, *p* = 0.0226). Baseline age, sex, disease duration, DAS28‐CRP, inflammatory markers (ESR, CRP), and seropositivity (RF/anti‐CCP Ab) were comparable between responders and nonresponders (all *p* > 0.05, Table 2). Given the exploratory nature and limited sample size of this subgroup, formal multivariable adjustment for established predictors of TNF inhibitor response was not performed.​ These exploratory findings suggest that Tph cells may be associated with ETN response and warrant validation in larger cohorts.

**TABLE 2:** Baseline characteristics of ETN‐treated patients by response status.

	ETN responders (*n* = 13)	ETN nonresponders (*n* = 12)	*p‐*value
General clinical data			
Age (years, mean ± SD)	45.54 ± 16.06	48.27 ± 12.51	0.65
Sex (*n*, %)			0.32
Male	4, 30.77	1, 8.33	∖
Female	9, 69.23	11, 91.67	∖
Disease duration (months, median [IQR])	36 (9.5, 85.5)	27 (12, 96)	0.73
Clinical characteristics			
TJC28 (median [IQR])	7 (3.5, 9)	6 (2, 20.75)	0.83
SJC28 (median [IQR])	3 (0.5, 5.5)	4 (2, 15.75)	0.24
PtGA VAS (10 cm, median [IQR])	5 (5, 8)	6 (5, 9.5)	0.32
DAS28‐CRP (mean ± SD)	4.23 ± 1.81	4.66 ± 2.02	0.58
Laboratory indicator			
WBC (×10^9^/L, mean ± SD)	6.81 ± 1.84	6.48 ± 1.67	0.65
HGB (g/L, mean ± SD)	131.91 ± 15.69	125.88 ± 15.52	0.06
PLT (×10^9^/L, mean ± SD)	294.67 ± 75.89	305.42 ± 97.99	0.76
ALT (U/L, mean ± SD)	17.23 ± 8.26	17.83 ± 9.78	0.91
AST (U/L, mean ± SD)	17.85 ± 8.20	18.17 ± 5.36	0.92
BUN (mmol/L, mean ± SD)	5.23 ± 1.87	5.74 ± 1.46	0.40
Cr (µmol/L, mean ± SD)	61.85 ± 16.53	65.67 ± 17.34	0.27
CRP (mg/L, median [IQR])	7.2 (0.7, 16.5)	7.3 (3, 41.2)	0.51
ESR (mm/h, median [IQR])	17.5 (14, 58)	24 (7.5, 80.5)	0.79
RF (IU/ml, median [IQR])	198.8 (52.15, 464.15)	18.75 (5.65, 136.5)	0.09
Anti‐CCP (RU/mL, mean ± SD)	174.53 ± 84.41	134.91 ± 95.05	0.28
Immune cell subsets, mean ± SD			
TNF‐α^+^Th cell (%)	37.67 ± 14.56	41.06 ± 4.76	0.47
IFN‐γ^+^Th cell (%)	18.12 ± 8.90	15.06 ± 3.77	0.30
IL‐2^+^Th cell (%)	47.73 ± 8.78	51.25 ± 9.44	0.37
Th17 cell (%)	1.18 ± 0.45	1.61 ± 0.44	0.06
CD161^+^Treg cell (%)	8.91 ± 4.96	7.86 ± 3.58	0.09
CLA^+^Treg cell (%)	16.33 ± 8.93	13.97 ± 10.26	0.56
Foxp3^+^Treg cell (%)	6.58 ± 1.86	5.68 ± 1.51	0.22
Teff cell (%)	90.32 ± 2.73	92.29 ± 1.98	0.06
Naïve Th cell (%)	37.72 ± 17.67	34.12 ± 10.40	0.56

*Note*: Normally distributed data were presented as mean ± standard deviation (SD). Nonnormally distributed data were presented as median [interquartile range, IQR]. Categorical variable was presented as frequencies and percentages.

Abbreviations: ALT, alanine aminotransferase; Anti‐CCP, anti‐cyclic citrullinated peptide antibody; AST, aspartate aminotransferase; BUN, blood urea nitrogen; Cr, creatinine; CRP, C‐reactive protein; ESR, erythrocyte sedimentation rate; ETN, etanercept; HGB, hemoglobin; PLT, platelets; PtGA, patients’ global assessment; RF, rheumatoid factor; SJC28, 28 swollen joint count; TJC28, 28 tender joint count; WBC, white blood cells.

**FIGURE 3:**
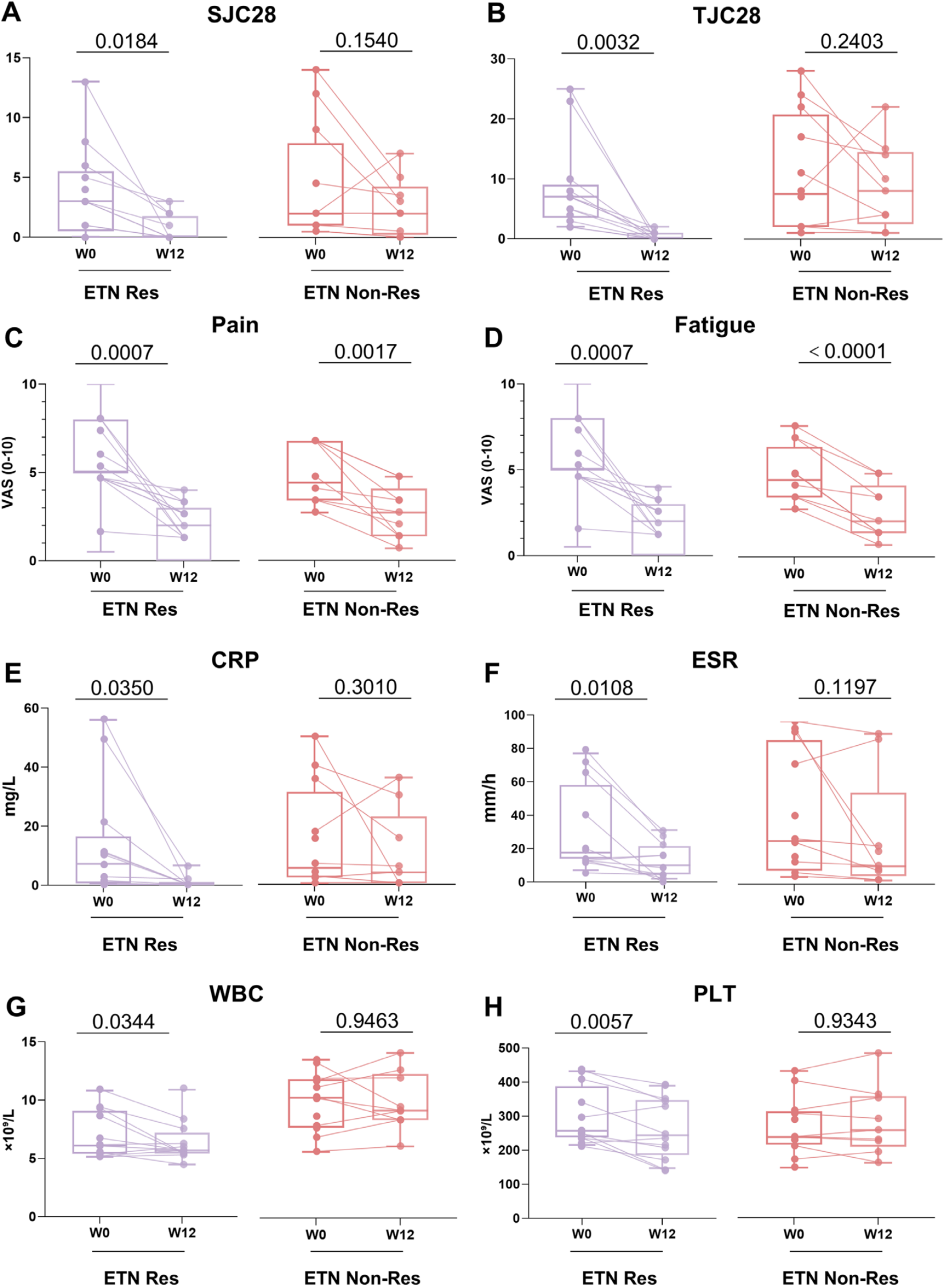
(A–H) Post‐treatment changes in clinical and laboratory indicators in ETN responders (*n* = 13) and nonresponders (*n* = 12). Significant reductions were observed in all parameters in responders (all *p* < 0.05), while no significant changes occurred in nonresponders. CRP, C‐reactive protein; ESR, erythrocyte sedimentation rate; ETN, etanercept; PLT, platelet count; SJC28, 28‐joint swollen joint count; TJC28, 28‐joint tender joint count; VAS, visual analog scale; WBC, white blood cell count.

**FIGURE 4:**
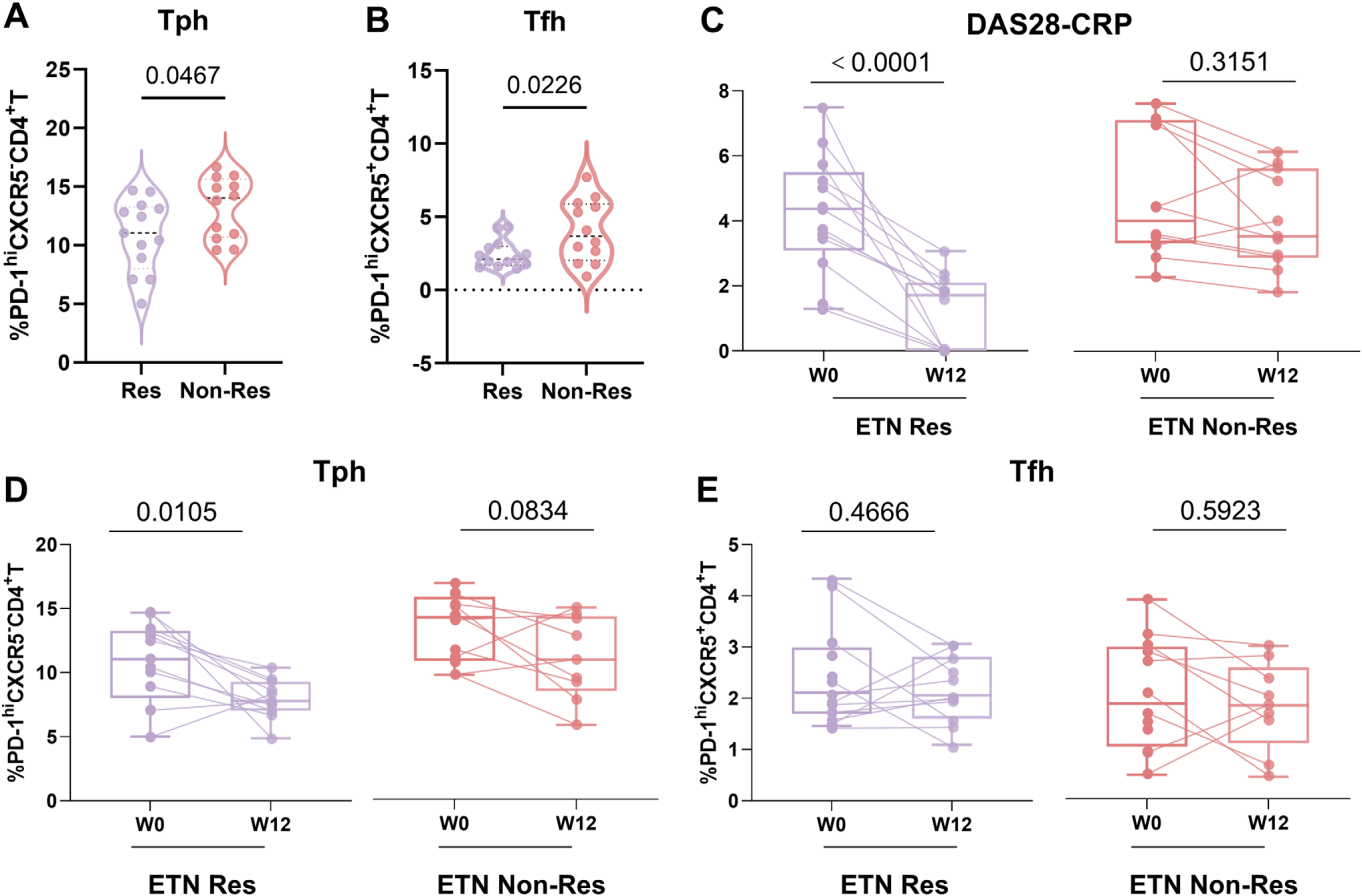
(A and B) At ETN treatment baseline, nonresponders (*n* = 12) exhibited a significantly higher percentage of Tph cells and Tfh cells compared to responders (*n* = 13). (C–E) ETN responders (*n* = 13) showed significant reductions in DAS28‐CRP and the percentage of Tph cells at Week 12, while Tfh cells remained stable. DAS28‐CRP, 8‐joint disease activity score based on C‐reactive protein; ETN, etanercept.

After 12 weeks of ETN treatment, significant reductions were observed in SJC28, TJC28, pain VAS, fatigue VAS, CRP, ESR, WBC, and PLT in responders, accompanied by a marked decrease in Tph cell frequency (Figure 3A–H). Baseline frequencies of Tph and Tfh cells were significantly higher in ETN nonresponders versus responders (Tph: *p* = 0.0467, Tfh: *p* = 0.0226). ​In responders, DAS28‐CRP scores decreased significantly from 4.23 ± 1.81 to 1.34 ± 1.13 (*p* < 0.0001), Tph cell frequency declined from 10.82 ± 3.08% to 7.97 ± 1.51% (*p* = 0.0105). However, Tfh cell frequency remained stable in both responders and nonresponders, with no statistically significant differences observed at week 12 (ETN responders: *p* = 0.4666, ETN nonresponders: *p* = 0.5923) (Figure 4A–E).

### 2.5 Serum IL‑21 Levels Reflected Tph Cell Function and Therapeutic Response

**FIGURE 5:**
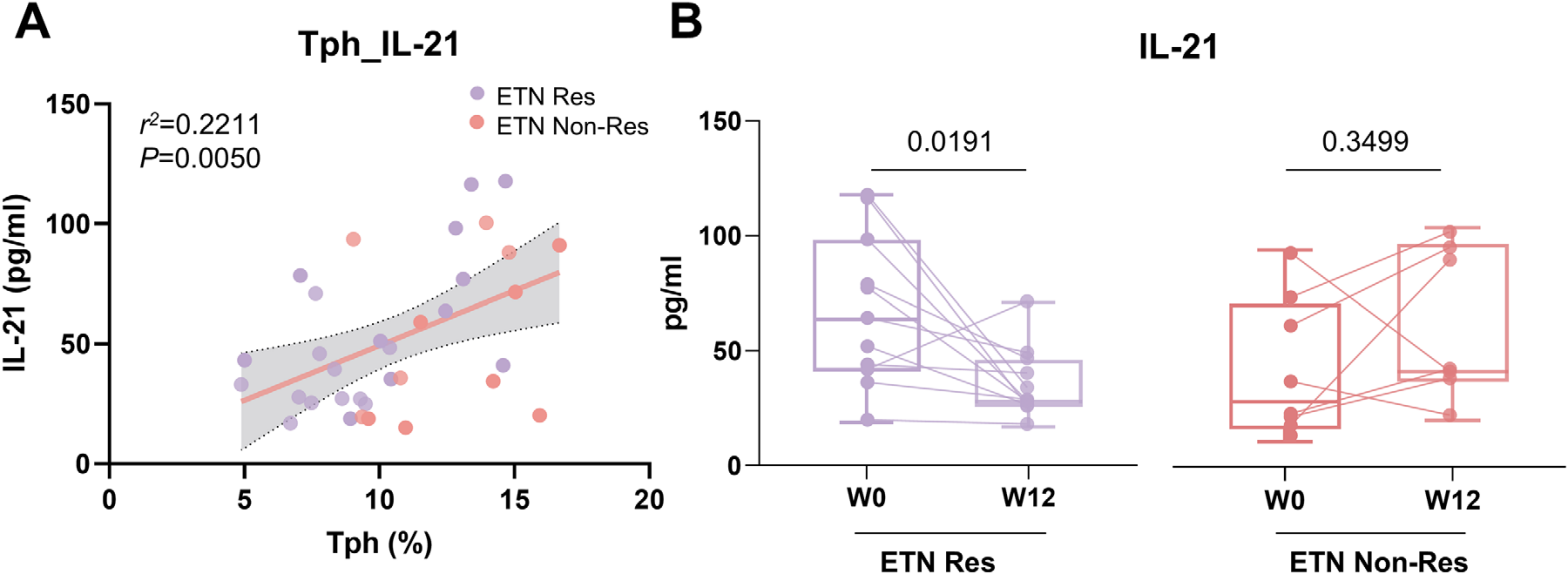
Relationship between circulating Tph cells and serum IL‐21, and longitudinal changes in IL‐21 during etanercept therapy. (A) Correlation between baseline serum IL‐21 levels (pg/mL) and circulating Tph cell frequency (%) in RA patients. Linear regression analysis was performed, with *r* ^2^ = 0.2211 and *p* = 0.0050. (B) Changes in serum IL‐21 concentrations (pg/mL) from baseline (W0) to week 12 (W12) in RA patients stratified by etanercept response status. Data are presented as box‐and‐whisker plots with individual paired patient trajectories. *p* values for within‐group changes were determined using paired Student's *t*‐tests (*p* = 0.0191 for responders, *p* = 0.3499 for nonresponders). ETN Non‐Res, etanercept nonresponders; ETN Res, etanercept responders.

At baseline, serum IL‑21 levels were positively correlated with circulating Tph cell frequency in RA patients (*r* ^2^ = 0.2211, *p* = 0.0050, Figure 5A). In the ETN responder group, serum IL‑21 levels showed a reduction from baseline (W0) to W12 (*p* = 0.0191, Figure 5B). In contrast, no significant change in IL‑21 levels was observed in the non‑responder group over the same period (*p* = 0.3499, Figure 5B).

Although the reduction in IL‑21 levels (ΔIL‑21) showed a positive trend with the reduction in Tph cell frequency (ΔTph), this correlation did not reach statistical significance (*r* ^2^ = 0.1047, *p* = 0.1418, Figure S6). Collectively, these findings support a potential functional link between Tph cells and IL‑21 production in RA.

## 3 Discussion

In this study, we observed a significant enrichment of Tph cells in the peripheral blood of RA patients. These cells demonstrated a strong association with RA disease activity and correlated with alterations in other immune cell subsets, suggesting a potential pathogenic role in RA. Furthermore, Tph cell frequency was independently associated with DAS28‐CRP score. A threshold of 9.41% optimally discriminated patients in remission from those with active disease, exhibiting high sensitivity for clinical assessment. While prior studies have established a correlation between Tph cells and disease activity [21, 22], our study provides a candidate cut‐off value for distinguishing disease activity states in this cohort, suggesting the potential of Tph cells as a novel biomarker for objective disease monitoring that warrants further validation.

Tph cells were initially identified in the synovium and synovial fluid of inflamed joints in RA by Rao et al. [14]. This cell subset exhibits a PD‐1^hi^CXCR5^−^CD4^+^ phenotype, implicating their involvement in the involvement in the formation of tertiary lymphoid structures. Through the secretion of IL‐21 and engagement of SLAMF5, Tph cells promote memory B‐cell differentiation and antibody production [23, 24]. In the present study, serum IL‐21 levels were positively correlated with Tph cell frequency at baseline and decreased significantly in ETN responders after 12 weeks of treatment. Furthermore, the reduction in IL‐21 was significantly correlated with the reduction in Tph cells, providing correlative functional evidence that supports the hypothesis that Tph cells mediate their pathogenic effects, at least in part, through IL‐21 secretion in RA. Subsequent studies have confirmed that Tph cells were also markedly enriched in the peripheral blood of seropositive RA patients, where they are thought to drive synovitis via the secretion of pro‐inflammatory cytokines [25]. Interestingly, despite their known helper function for B cells, we observed no significant correlation between Tph cell levels and autoantibody (RF or Anti‐CCP) titers. This dissociation suggests that Tph cells may contribute predominantly to synovitis inflammation rather than to autoantibody production itself. While RF and ACPA are valuable diagnostic markers, monitoring Tph cell dynamics provides an objective reflection of disease activity. Their combined use could therefore bridge the gap between diagnosis and ongoing disease activity assessment in RA.

We further evaluated the relationship between Tph cells and other immune cell subsets in RA. Among these subsets, Tph cell frequency showed a positive correlation with Tfh cells. Tfh cells are characterized by PD‐1^hi^CXCR5^+^ phenotype [26]. Their key distinction from Tph cells lies in the differential expression of CXCR5, which directs Tfh cell migration to germinal centers within secondary lymphoid organs (SLOs), whereas Tph cells primarily reside at sites of inflammation. However, some studies hypothesize that these two subsets may share a common origin and exhibit bidirectional plasticity. The study by Fortea‐Gordo et al. [27] similarly observed elevated Tfh cells in the peripheral blood of RA patients, with only minimal infiltration in the synovium compared to their predominant presence in SLOs. Current evidence suggests complementary roles of Tfh and Tph cells in RA pathogenesis: Tfh cells initiate immune responses via autoantibody production in SLOs during early disease phase, while Tph cells perpetuate synovial inflammation during disease progression. The phenotypic and functional similarities between these subsets, coupled with their synergistic contributions to RA pathogenesis, likely explain the positive correlation between Tph and Tfh cells observed in our study [28, 29].

Although TNF‐αi therapy is widely used in RA with demonstrated efficacy, approximately 20%–40% of patients exhibit an inadequate treatment response [6]. The mechanisms underlying this nonresponse remain unclear, and current clinical biomarkers offer limited predictive value for treatment outcomes. Julià et al. employed a cell‐deconvolution approach and reported that a lower abundance of Tph cells in the synovium of RA patients at baseline was associated with a favorable response to TNF‐αi therapy [30]. However, the relationship between circulating Tph cells and treatment response remains unclear.

In our study, in an exploratory analysis, we demonstrated that RA patients with a lower frequency of peripheral blood Tph cells at baseline tended to have a more favorable response to ETN therapy. Given the small sample size and lack of multivariate adjustment, this association should be considered preliminary and hypothesis‐generating rather than independently predictive. Although​ baseline clinical characteristics, including those listed, were comparable between responders and nonresponders, the limited sample size precluded a formal multivariable analysis to ascertain the independent predictive value of Tph cells. Meanwhile, Tfh cells showed a similar predictive pattern. While TNF‐αi thereby effectively reduces TNF‐α levels and blocks receptor signaling to alleviate synovitis, Tph cells may sustain disease progression through direct involvement in synovial inflammation and alternative cytokine networks. This may partly explain the inadequate treatment response observed in patients with elevated Tph cells.

We also examined longitudinal changes after 12 weeks therapy and found that in responders, Tph cell frequency decreased significantly in parallel with the reduction in DAS28‐CRP. In contrast, no significant changes in Tph cells or DAS28‐CRP were observed in nonresponders. Meanwhile, Tfh cell levels remained stable in both responders and nonresponders throughout the follow‐up period. These findings align with the report by Fortea‐Gordo et al., implying that Tfh cells may represent a stable immunological aberration in RA, reflecting fundamental immune dysregulation [27]. In comparison, Tph cells appear to reflect dynamic systemic immune activation, with their fluctuations correlating with disease activity and local inflammatory status. Together, these exploratory findings support the potential utility of Tph cells as a candidate dynamic biomarker for monitoring disease progression and therapeutic response in RA, which requires validation in larger cohorts.

Several limitations of this study should be acknowledged. First, we did not perform in vitro co‐culture experiments, so the precise mechanisms by which Tph cells drive RA pathogenesis remain to be elucidated. Second, the modest sample size (*n* = 58 for cross‐sectional analysis; *n* = 25 for the ETN subgroup) may reduce statistical power and increase the risk of overfitting, particularly given that the multivariable regression model included multiple immune cell subsets. The single‑cohort design also precludes internal and external validation, and the candidate Tph cut‑off value (9.41%) is exploratory rather than validated. Third, the ETN‑treated subgroup was small, and we could not adjust for all established clinical predictors of TNF inhibitor response. Consequently, the observed associations, including the cut‑off value and the ETN response finding, are hypothesis‑generating and require validation in larger, independent, prospective cohorts.

## 4 Conclusions

Our study demonstrated that circulating Tph cells were significantly elevated in RA patients and exhibited a strong positive correlation with disease activity, supporting their potential involvement in RA pathogenesis. In this exploratory, single‐center cohort, a candidate Tph cell threshold of 9.41% showed preliminary value in distinguishing disease activity states, and baseline Tph levels were associated with clinical response to ETN in a hypothesis‑generating analysis. However, these findings are derived from a limited sample without external validation, the cut‑off is prone to overfitting, and the predictive relationship requires confirmation.​ Therefore, Tph cells may represent a candidate​ biomarker for disease activity monitoring and a potential​ predictor of treatment response, but their clinical utility must be prospectively validated in larger, independent cohorts.

## 5 Methods

### 5.1 Patients and Ethics Statement

Fifty‐eight RA patients were enrolled in this study from the Rheumatology Department of Peking University People's Hospital from July 2022 to September 2024. All patients fulfilled the 2010 American College of Rheumatology (ACR)/European League Against Rheumatism (EULAR) RA classification criteria [31]. This was a real‐world clinical cohort recruited during routine clinical practice, encompassing a spectrum of disease activity and treatment status. In statistical analysis, we focused on the association of Tph cells with disease activity across the full spectrum of RA severity. Treatment‐stratified comparisons were restricted to subgroups with sufficient sample size. The ETN‐treated subgroup was analyzed separately as a clinically homogeneous population.

For comparison, 12 age‐ and sex‐matched OA patients and 15 HC were included. Exclusion criteria for all participants were acute or chronic infections, malignancies, hematologic disorders, and other autoimmune or autoinflammatory diseases. For OA patients, additional exclusions were applied to minimize immune‐related confounding. Individuals were excluded if they had any other rheumatic and autoimmune diseases, clinically significant chronic inflammatory disorders, primary immune dysfunction, acute or chronic infection, malignancy, or severe organ dysfunction.

Among the RA patients, 25 with active disease and inadequate response to csDMARDs were initiated on subcutaneous ETN at a dosage of 25 mg twice weekly and were followed for 12 weeks. Disease activity was assessed primarily using the DAS28‐CRP. According to DAS28‐CRP scores, disease activity was categorized into complete remission (DAS28‐CRP ≤ 2.6), low (2.6<DAS28‐CRP ≤ 3.2), moderate (3.2<DAS28‐CRP ≤ 5.1), and high (DAS28‐CRP > 5.1) [32]. Clinical response to ETN was defined using ACR20 criteria, which utilizes 28‑joint counts for tender and swollen joints [33]. Patients who failed to achieve an ACR20 response at week 12 were defined as nonresponders, and all others were classified as responders [34].

**FIGURE 6:**
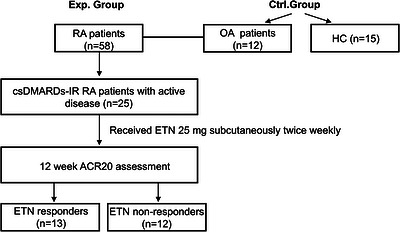
A flowchart of the study design. ACR 20 response, American College of Rheumatology 20% Response Criteria; csDMARDs‐IR, inadequate response to conventional synthetic disease‐modifying antirheumatic drugs; ETN, etanercept; HC, healthy controls; OA, osteoarthritis; RA, rheumatoid arthritisTo meet this criterion, patients must achieve ≥ 20% improvement in both swollen and tender joint counts, as well as ≥20% improvement in at least one of the following three domains: patient‐reported pain score, patient/physician global assessment, and inflammatory markers (C‐reactive protein [CRP] or erythrocyte sedimentation rate [ESR]).

The study protocol was approved by the Institutional Review Board of Peking University People's Hospital (2021PHB452‐001). Written informed consent was obtained from all RA and OA patients as well as from the HC. A flowchart of the study design is presented in Figure 6.

### 5.2 Data Collection

Clinical and laboratory data from all patients were extracted from the Computerized Patient Record System. For RA patients, collected demographic data included age, sex, disease duration, smoking history, prior treatments, and the presence of extra‐articular systemic involvement. Clinical characteristics comprised the swollen joint count (SJC) and tender joint count (TJC) based on a 28‐joint assessment, patient global assessment visual analog scale (PtGA VAS), evaluator global assessment VAS (EGA VAS), pain VAS, fatigue VAS, and the DAS28‐CRP. Laboratory parameters consisted of WBC, neutrophils (Neu), lymphocytes (Lym), monocytes (Mo), HGB, PLT, ALT, AST, BUN, Cr, CRP, and ESR. RF and anti‐CCP Ab levels were measured by enzyme‐linked immunosorbent assay (ELISA).

### 5.3 Flow Cytometry

Flow cytometry analysis was performed to characterize immune cell subsets. The specific surface markers used to define each cell population are listed in Table S4. Peripheral blood samples were collected in EDTA‑anticoagulated tubes and processed within 4 h at room temperature to minimize artifact for sensitive markers including PD‑1 and chemokine receptors. Peripheral blood mononuclear cells (PBMCs) were isolated by Ficoll‑Hypaque density gradient centrifugation according to standard protocols. Briefly, whole blood was diluted with phosphate‑buffered saline (PBS), carefully overlaid onto Ficoll solution, and centrifuged at 1800 rpm for 20 min without braking. PBMCs were then collected, washed twice in PBS, and resuspended in staining buffer for subsequent immunofluorescence staining. Cells were stained with fluorophore‑conjugated monoclonal antibodies against human CD3, CD4, CD45RA, CXCR5, PD‐1, CD25, Foxp3, CD127, cutaneous lymphocyte antigen (CLA), CD161, CD8, CD56, CD16, and CD19. All antibodies were purchased from BD Biosciences and BioLegend (San Diego, CA, USA), and details are provided in Table S5. Tph cells were defined as PD‑1^hi^CXCR5^−^CD4^+^ T cells. Gating was performed sequentially: lymphocytes → single cells → CD3^+^CD4^+^ T cells → PD‑1^hi^CXCR5^−^ cells. Tph cell frequency was calculated as the percentage of CD4^+^ T cells (Figure S7). Fluorescence minus one control were used to ensure accurate gating for PD‑1 and CXCR5. Flow cytometric data were acquired on a Beckman Coulter CytoFLEX flow cytometer (Beckman Coulter, Brea, CA, USA). Data acquisition and analysis were performed with CytoExpert software (Version 2.4, Beckman, CA, USA).

### 5.4 Elisa

Serum IL‐21 concentrations were measured using a commercial human ELISA kit according to the manufacturer's instructions (Lianke Bio, China). Peripheral venous blood samples were collected at baseline and week 12 after ETN treatment, clotted at room temperature, and centrifuged at 3000 rpm for 10 min to obtain serum. Serum samples were stored at −80°C until analysis. The absorbance at 450 nm was measured using a Synergy 2 Multi‐Mode Microplate Reader (BioTek Instruments, Winooski, VT, USA). IL‐21 concentrations were calculated according to a standard curve prepared with serial dilutions of recombinant human IL‐21. All samples and standards were measured in duplicate to ensure reproducibility.

### 5.5 Statistical Analysis

Statistical analyses were conducted using SPSS Statistics (Version 25.0, IBM Corp., Armonk, NY, USA) and GraphPad Prism (Version 10, GraphPad Software, San Diego, CA, USA), with additional analyses conducted in Zstats and R software (Version 4.4.0). Normally distributed data was presented as mean ± standard deviation (SD), while nonnormally distributed data was presented as median with interquartile range (IQR). For categorical variables, frequencies and percentages were used for descriptive analysis. Between‑group comparisons used independent *t*‑test (two groups) for normal continuous variables, Mann‐‑Whitney *U* test (two groups) or Kruskal‐‑Wallis *H* test (multiple groups) for non‑normal variables. Bivariate correlations used Pearson or Spearman tests. Binary logistic regression was used for univariate and multivariate analyses. Variable selection was based on biological relevance; Tph cells were the pre‑specified primary variable of interest. Given the modest sample size, we explicitly acknowledge the exploratory nature of the regression and avoid overinterpretation. Multicollinearity was assessed by variance inflation factors (all VIF < 2). ROC curves were plotted and AUC was calculated; the Youden index identified the optimal cut‑off for stratification. To reduce false‑positive risk, multiple testing was restricted to pre‑specified immunological markers. Baseline characteristics, between‑group differences, and potential confounders (including disease activity and technical batch effects) were systematically evaluated. A two‑sided *p* < 0.05 was considered statistically significant.

## Author Contributions

Haojie Xu, Wanki Ho, Lulu Cao, and Huaqun Zhu performed most of the data collection and drafted the manuscript. Zhanguo Li, Huaqun Zhu, and Hua Ye conceived the study and participated in the design and interpretation of results. Ru Li helped to collect samples. Dongdong Fu, Yun Li, Feng Sun, and Xi Xu participated in the immune cell detection. All the authors read and approved the final manuscript.

## Funding

This work was supported by the National Natural Science Foundation of China (No. 82402103 to Dr. HQ Zhu, No. 92374202 to Prof. ZG Li), the Major Special Project of the National Health Commission of the People's Republic of China (No. 2025ZD18010 to Dr. HQ Zhu), and the Research and Development Foundation of Peking University People's Hospital (No. RDx2025‐03, No. RDL2024‐17, and No. RDLX2023‐01 to Dr. HQ Zhu).

## Ethics Statement

This study was approved by the Institutional Review Board of Peking University People's Hospital (2021PHB452‐001).

## Conflicts of Interest

The authors declare no conflicts of interest.

## Supporting information




**Supplementary File1: Table S1**: Demographic and clinical characteristics of RA patients. **Table S2**: Baseline demographic characteristics of study groups. **Table S3**: (A) Univariate assessment of the association between immune cell subsets and DAS28‐CRP scores. (B) Multivariable assessment of the association between immune cell subsets and DAS28‐CRP scores. **Table S4**: Markers and representation of immune cell subsets. **Table S5**: Flow cytometry fluorescent antibodies. **Figure S1**: Subgroup analysis of Tph cell frequency stratified by csDMARDs regimens. **Figure S2**: The percentage of Tph cells was positively correlated with (A) WBC and (B) PLT, while negatively correlated with (C) HGB. **Figure S3**: Correlation analysis of Tph cell frequency with clinical and laboratory parameters. (A) age, (B) duration, (C) Sex, (D) smoking, (E) alcohol, (F) alanine transaminase (ALT), (G) blood urea nitrogen (BUN), (H) creatinine (Cr), (I) rheumatoid factor (RF), (J) anti‐cyclic citrullinated peptide antibody (Anti‐CCP Ab). **Figure S4**: Correlations between Tph cells and additional composite disease activity indices. (A) Correlation between Tph cell frequency and continuous CDAI. (B) Correlation between Tph cell frequency and continuous SDAI. (C) Progressive increase in Tph cell levels across four‐category CDAI classification. (D) Progressive increase in Tph cell levels across four‐category SDAI classification. **Figure S5**: Receiver operating characteristic curve and area under the curve (AUC) value of Tph cells. **Figure S6**: Correlation between changes in serum IL‑21 levels (ΔIL‑21) and changes in circulating Tph cell frequency (ΔTph) in RA patients following etanercept treatment. **Figure S7**: Gating strategy: (1) Tph cell (PD‑1^hi^CXCR5^−^CD4^+^ T cell). (2) Tfh cell (PD‑1^hi^CCR7^+^CXCR5^+^CD4^+^ T cell); Naïve Th cell (CD3^+^CD4^+^CD45RA^+^). (3) Foxp3^+^ Treg cell (CD3^+^CD4^+^CD25^hi^Foxp3^+^); Teff cell (CD3^+^CD4^+^CD25^low^Foxp3^−^). (4) CLA^+^Treg cell (CLA^+^CD25^hi^CD127^low^T cell); CD161^+^Treg cell (CD161^+^ CD25^hi^CD127^low^T cell). (5) TNF‐α^+^Th cell; IFN‐γ^+^Th cell; IL‐2^+^Th cell; Th17 cell.

## Data Availability

The data supporting the findings of this study are available from the corresponding author upon reasonable request.
