# Supplementary material for: PD‐1hiCXCR5−CD4+T Peripheral Helper Cells Were Enriched and Potentially Predicted Clinical Response to Etanercept Therapy in Rheumatoid Arthritis
Source: MedComm (2020). 2026 Jul 30;7(8):e70876. doi: 10.1002/mco2.70876 (PMC13420207; doi:10.1002/mco2.70876)

**PD-1^hi^CXCR5^-^CD4^+^T Peripheral Helper Cells were Enriched and Potentially Predict****ed Clinical Response to** **Etanercept Therapy in Rheumatoid Arthritis**

Haojie Xu^1#^, Wanki Ho^1#^, Lulu Cao^1#^, Dongdong Fu^1^, Yun Li^1^, Feng Sun^1^, Xi Xu^1^, Zhanguo Li^1^, Ru Li^1^, Hua Ye^1*^, Huaqun Zhu^1*^

^1^Department of Rheumatology and Immunology, Peking University People’s Hospital, Beijing, China. & Beijing Key Laboratory of Non-invasive Diagnosis and Immunotherapy of Rheumatic Diseases, Beijing, China.

^#^Haojie Xu, Wanki Ho and Lulu Cao contributed equally to this work.

***Corresponding author:**

Department of Rheumatology and Immunology, Peking University People’s Hospital, 11 Xizhimen South Street, Xicheng District, Beijing, 100044, China. E-mails: [zhuhuaqun@126.com](mailto:zhuhuaqun@126.com) to Dr. Huaqun Zhu, [yehbmu@126.com](mailto:yehbmu@126.com) to Prof. Hua Ye.

**Supplementary Tables**

**Table S1.** Demographic and clinical characteristics of RA patients

| **Variable​** | **Value​** |
| --- | --- |
| **General Clinical Data**​ |  |
| Age, years (Mean±SD) | 51.31±14.17 |
| Female, n (%) | 47 (81.0) |
| Disease duration, months (Median [IQR]) | 48 (12, 122) |
| ILD, n (%) | 4 (6.9) |
| Smoking history, n (%) | 9 (15.5) |
| Alcohol consumption, n (%) | 7 (12.1) |
| **Clinical Characteristics**​ |  |
| TJC28 (Median [IQR]) | 2.5 (0.75, 7.25) |
| SJC28 (Median [IQR]) | 2 (0, 4) |
| PtGA VAS (10 cm, Median [IQR]) | 5 (2, 6) |
| EGA VAS (10 cm, Median [IQR]) | 5 (2.75, 8) |
| Pain VAS (10 cm, Median [IQR]) | 4 (1, 6) |
| Fatigue VAS (10 cm, Median [IQR]) | 5 (2, 6) |
| DAS28-CRP (Mean±SD) | 3.58±2.03 |
| CDAI (Median [IQR]) | 14.5 (5.75, 27) |
| SDAI (Median [IQR]) | 21.45 (7.98, 55.58) |
| **Laboratory Indicators**​ |  |
| WBC (×10⁹/L) (Mean±SD) | 6.71±2.11 |
| Neu (%) (Mean±SD) | 66.07±10.25 |
| Lym (%) (Mean±SD) | 25.22±9.60 |
| Mo (%) (Mean±SD) | 6.37±1.96 |
| HGB (g/L) (Mean±SD) | 124.04±18.12 |
| PLT (×10⁹/L) (Mean±SD) | 285.46±93.84 |
| ALT (U/L) (Mean±SD) | 19.29±13.63 |
| AST (U/L) (Mean±SD) | 20.43±8.25 |
| Urea (mmol/L) (Mean±SD) | 5.18±1.43 |
| Cr (μmol/L) (Mean±SD) | 59.1±15.52 |
| CRP (mg/L) (Median [IQR]) | 3.4 (0.5, 23.57) |
| ESR (mm/h) (Median [IQR]) | 17.5 (10.75, 50.5) |
| RF (IU/ml) (Median [IQR]) | 63.1 (11.5, 269.63) |
| Anti-CCP (RU/ml) (Median [IQR]) | 174.17 (82.74, 240.72) |
| **Previous Treatment**​ |  |
| Untreated, n (%) | 3 (5.2) |
| csDMARDs-only, n (%) | 25 (43.1) |
| cs+bDMARDs, n (%) | 28 (48.3) |
| cs+tsDMARDs, n (%) | 2 (3.4) |

Note:​ Data are presented as mean ± standard deviation (SD), median (interquartile range, IQR), or number (percentage) as appropriate.

Abbreviations: RA, rheumatoid arthritis; ILD, interstitial lung disease; TJC28, 28-joint tender joint count; SJC28, 28-joint swollen joint count; PtGA, patient's global assessment; EGA, evaluator's global assessment; VAS, visual analog scale; DAS28-CRP, Disease Activity Score 28 based on C-reactive protein; CDAI, Clinical Disease Activity Index; SDAI, Simplified Disease Activity Index; WBC, white blood cell count; Neu, neutrophil percentage; Lym, lymphocyte percentage; Mo, monocyte percentage; HGB, hemoglobin; PLT, platelet count; ALT, alanine aminotransferase; AST, aspartate aminotransferase; Cr, creatinine; CRP, C-reactive protein; ESR, erythrocyte sedimentation rate; RF, rheumatoid factor; Anti-CCP, anti-cyclic citrullinated peptide; csDMARDs, conventional synthetic disease-modifying antirheumatic drugs; bDMARDs, biologic disease-modifying antirheumatic drugs; tsDMARDs, targeted synthetic disease-modifying antirheumatic drugs.

**Table S2.** Baseline demographic characteristics of study groups

| **Characteristic** | **RA (n=58)** | **OA (n=12)** | **HC (n=15)** | ***P-value*** |
| --- | --- | --- | --- | --- |
| Age | 51.31±14.17 | 56.25±9.68 | 56.33±9.34 | >0.05 |
| Female, n (%) | 47, 81% | 10, 83.33% | 12, 80% | >0.05 |

Abbreviations: RA, rheumatoid arthritis; OA, osteoarthritis; HC, healthy controls.

**Table S3. (A)** Univariate assessment of the association between immune cell subsets and DAS28-CRP scores

| Variable | Univariate binary logistic regression analysis | | | | |
| --- | --- | --- | --- | --- | --- |
|  | β | SE | Wald χ² | P | OR, 95% CI |
| **Tph (%)** | **0.54** | **0.15** | **3.55** | **<0.001** | **1.72(1.28~2.32)** |
| TNF-α⁺Th (%) | -0.06 | 0.03 | -1.90 | 0.058 | 0.94(0.88~1.00) |
| IFN-γ⁺Th (%) | -0.08 | 0.04 | -1.83 | 0.067 | 0.92(0.85~1.01) |
| IL-2⁺Th (%) | -0.01 | 0.03 | -0.29 | 0.773 | 0.99(0.93~1.05) |
| Th17 (%) | -0.47 | 0.54 | -0.88 | 0.377 | 0.62(0.22~1.78) |
| CD161⁺Treg (%) | -0.03 | 0.05 | -0.65 | 0.513 | 0.97(0.88~1.07) |
| Foxp3⁺ (%) | 0.09 | 0.20 | 0.44 | 0.661 | 1.09(0.74~1.61) |
| Teff (%) | -0.07 | 0.14 | -0.52 | 0.602 | 0.93(0.71~1.22) |
| Naïve Th (%) | 0.01 | 0.03 | 0.41 | 0.683 | 1.01(0.96~1.06) |
| Tfh (%) | 0.24 | 0.18 | 1.36 | 0.175 | 1.28(0.90~1.82) |

Abbreviations: Tph cells, T peripheral helper cells; Tfh, T follicular helper cells; β, regression coefficient; SE, standard error; OR, odds ratio; CI, confidence interval; SE, standard error. *P* < 0.05 was considered statistically significant.

**(B)** Multivariable assessment of the association between immune cell subsets and DAS28-CRP scores

| Variable | Multivariable binary logistic regression analysis | | | | |
| --- | --- | --- | --- | --- | --- |
|  | β | SE | Wald χ² | P | OR, 95% CI |
| **Tph (%)** | **0.75** | **0.37** | **2.05** | **0.040** | **2.12(1.03~4.37)** |
| TNF-α⁺Th (%) | -0.00 | 0.07 | -0.03 | 0.976 | 1.00(0.87~1.15) |
| IFN-γ⁺Th (%) | 0.04 | 0.10 | 0.42 | 0.673 | 1.04(0.85~1.28) |
| IL-2⁺Th (%) | -0.01 | 0.09 | -0.12 | 0.904 | 0.99(0.82~1.19) |
| Th17 (%) | -1.87 | 1.14 | -1.63 | 0.103 | 0.15(0.02~1.45) |
| CD161⁺Treg (%) | -0.02 | 0.10 | -0.23 | 0.818 | 0.98(0.81~1.18) |
| Foxp3⁺ (%) | -0.45 | 0.69 | -0.66 | 0.512 | 0.63(0.16~2.47) |
| Teff (%) | 0.18 | 0.38 | 0.47 | 0.637 | 1.20(0.57~2.53) |
| Naïve Th (%) | -0.07 | 0.07 | -0.95 | 0.344 | 0.93(0.81~1.08) |
| Tfh (%) | 0.25 | 0.42 | 0.60 | 0.546 | 1.29(0.57~2.91) |

Abbreviations: Tph cells, T peripheral helper cells; Tfh, T follicular helper cells; β, regression coefficient; SE, standard error; OR, odds ratio; CI, confidence interval; SE, standard error. *P* < 0.05 was considered statistically significant.

**Table S4.** Markers and representation of immune cell subsets

| **Immune cell subsets** | **Surface markers** |
| --- | --- |
| T cell | CD3^+^ |
| CD4^+^T cell | CD3^+^CD4^+^CD8^-^ |
| Naive Th cell | CD3^+^CD4^+^CD45RA^+^ |
| TNF-α^+^Th cell | CD3^+^CD4^+^TNF-α^+^ |
| IFN-γ^+^Th cell | CD3^+^CD4^+^IFN-γ^+^ |
| IL-2^+^Th cell | CD3^+^CD4^+^IL-2^+^ |
| Th17 cell | CD3^+^CD4^+^IL-17A^+^ |
| Teff cell | CD3^+^CD4^+^Foxp3^-^CD25^hi^ |
| Treg cell | CD3^+^CD4^+^Foxp3^+^CD25^hi^ |
| CD161^+^Treg cell | CD3^+^CD4^+^CD161^+^CD25^hi^CD127^low^ |
| Tfh cell | CD3^+^CD4^+^CD45RA^-^PD1^+^CXCR5^+^ |
| Tph cell | CD3^+^CD4^+^CD45RA^-^PD1^+^CXCR5^-^ |

**Table S5.** Flow cytometry fluorescent antibodies

|  | **Catalog Number** | **Antibody Target** | **Fluorophore** |
| --- | --- | --- | --- |
| **Th1/Th2/Th17** **Cell Staining Panel** | | | |
| BD Biosciences (USA) | 652831 | CD3 | PerCP |
|  | 340443 | CD4 | APC |
|  | 340448 | IL-2 | FITC |
|  | 340451 | IL-4 | PE |
|  | 566736 | IL-17A | PE |
|  | 340449 | IFN-γ | FITC |
|  | 340512 | TNF-α | PE |
| **Foxp3^+^Treg & Teff Cell Staining Panel** | | | |
| BD Biosciences (USA) | 652831 | CD3 | PerCP |
|  | 340133 | CD4 | FITC |
|  | 341009 | CD25 | PE |
|  | 560045 | Foxp3 | AF647 |
| **Tph/Tfh & naïve Th Cell Staining Panel** | | | |
| BD Biosciences (USA) | 641397 | CD3 | APC-H7 |
|  | 340133 | CD4 | FITC |
|  | 563031 | CD45RA | BV510 |
|  | 561272 | PD-1 | PE-CY7 |
|  | 558113 | CXCR5 | AF647 |

**Supplementary Figures**

**Figure S1.** Subgroup analysis of Tph cell frequency stratified by csDMARDs regimens


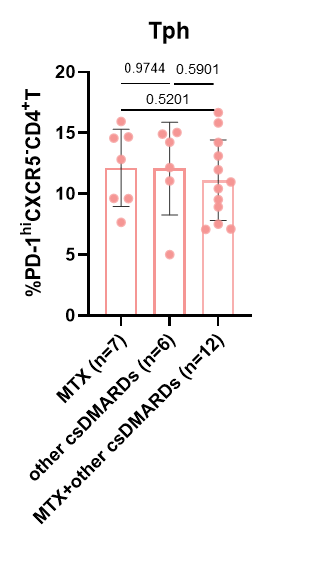


Abbreviations: Tph cells, T peripheral helper cells; MTX, methotrexate; csDMARDs, conventional synthetic disease-modifying antirheumatic drugs; bDMARDs, biologic disease-modifying antirheumatic drugs; tsDMARDs, targeted synthetic disease-modifying antirheumatic drugs.

**Figure S2.** The percentage of Tph cells was positively correlated with (A) WBC and (B) PLT, while negatively correlated with (C) HGB.


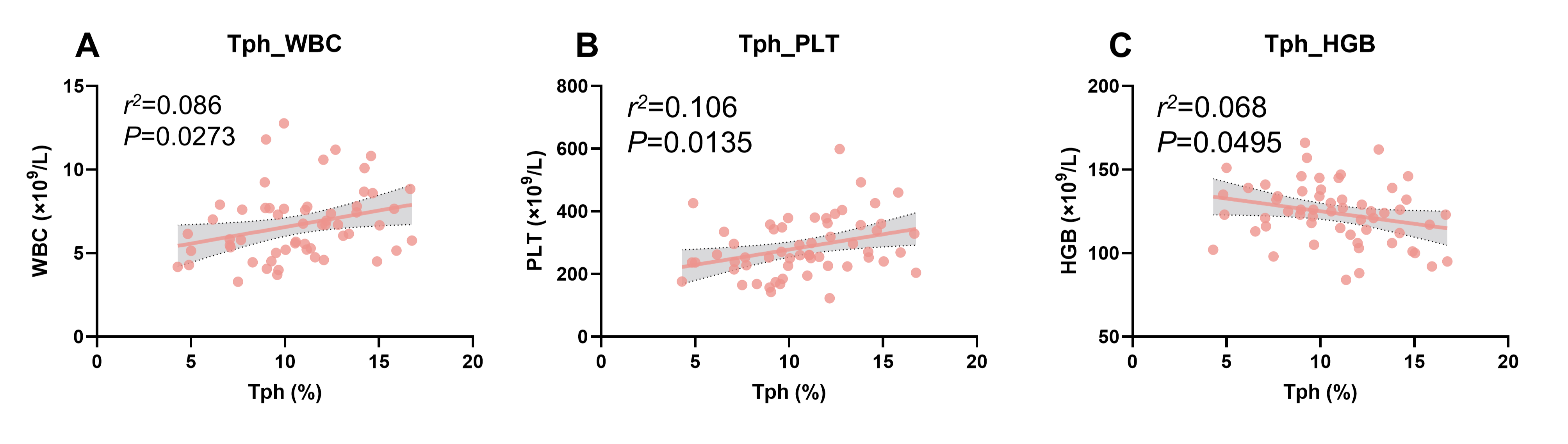


Abbreviations: Tph cells, T peripheral helper cells; WBC, white blood cell count; PLT, platelet count; HGB, hemoglobin.

**Figure S3.** Correlation analysis of Tph cell frequency with clinical and laboratory parameters


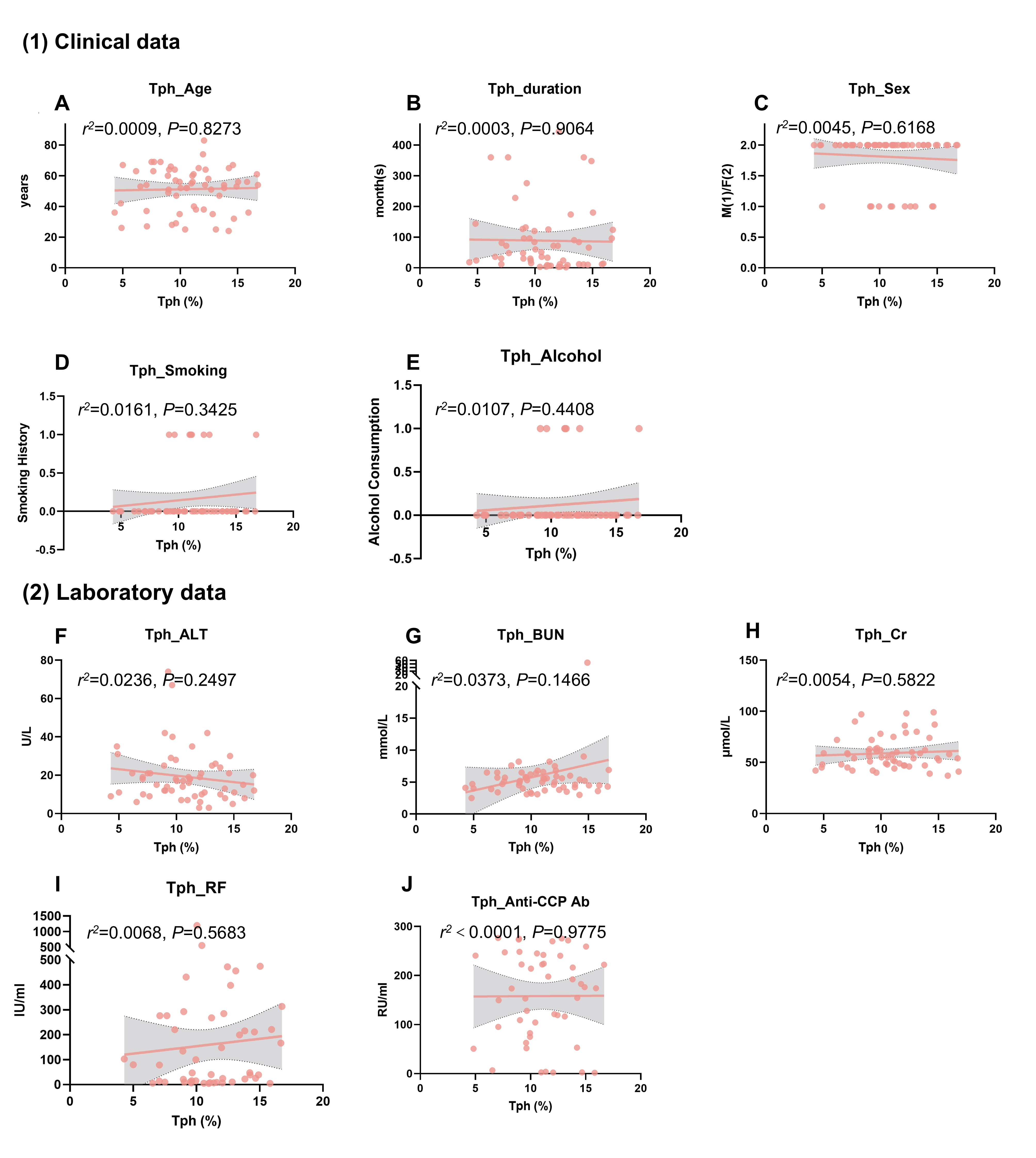


Abbreviations: Tph cells, T peripheral helper cells; ALT, alanine transaminase; BUN, blood urea nitrogen; Cr, creatinine; RF, rheumatoid factor; Anti-CCP Ab, anti-cyclic citrullinated peptide antibody.

**Figure S4.** Correlations between Tph cells and additional composite disease activity indices.

(A) Correlation between Tph cell frequency and continuous CDAI. (B) Correlation between Tph cell frequency and continuous SDAI. (C) Progressive increase in Tph cell levels across four-category CDAI classification. (D) Progressive increase in Tph cell levels across four-category SDAI classification.

**
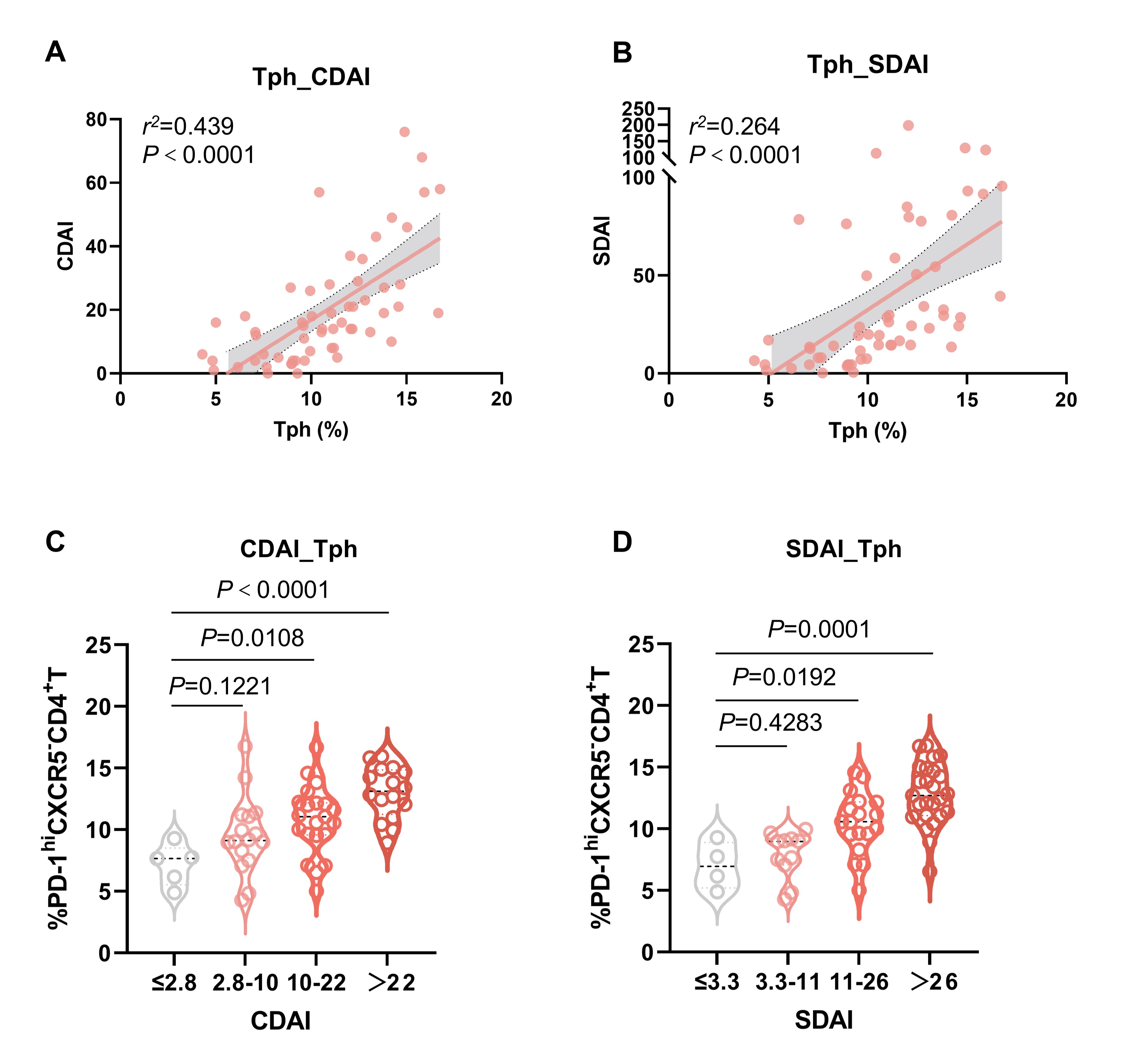
**

Abbreviations: Tph cells, T peripheral helper cells; CDAI, clinical disease activity index; SDAI, simplified disease activity index.

**Figure S5.** Receiver operating characteristic curve and area under the curve (AUC) value of Tph cells.


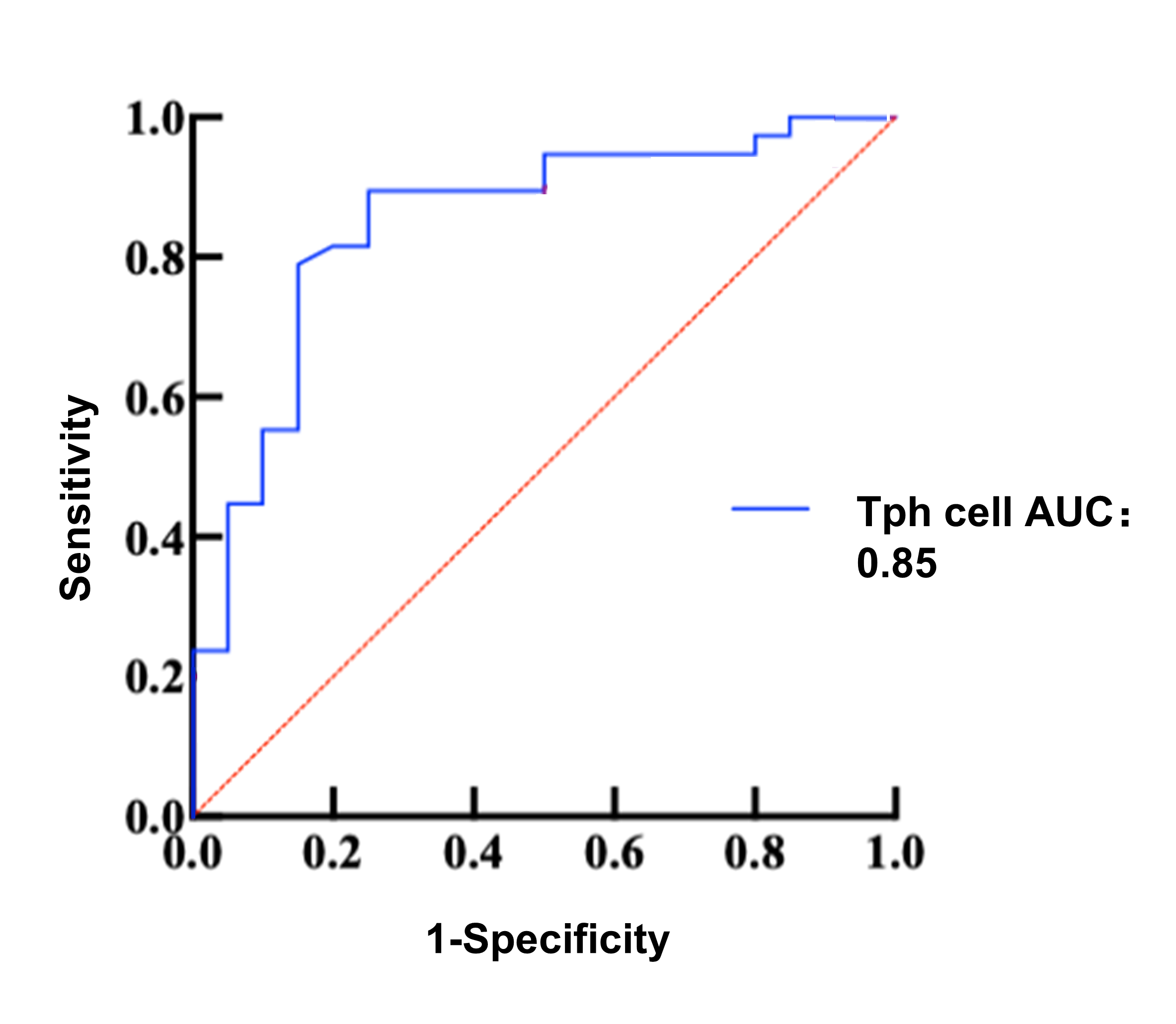


Abbreviations: Tph cells, T peripheral helper cells.

**Figure S6.** Correlation between changes in serum IL‑21 levels (ΔIL‑21) and changes in circulating Tph cell frequency (ΔTph) in RA patients following etanercept treatment


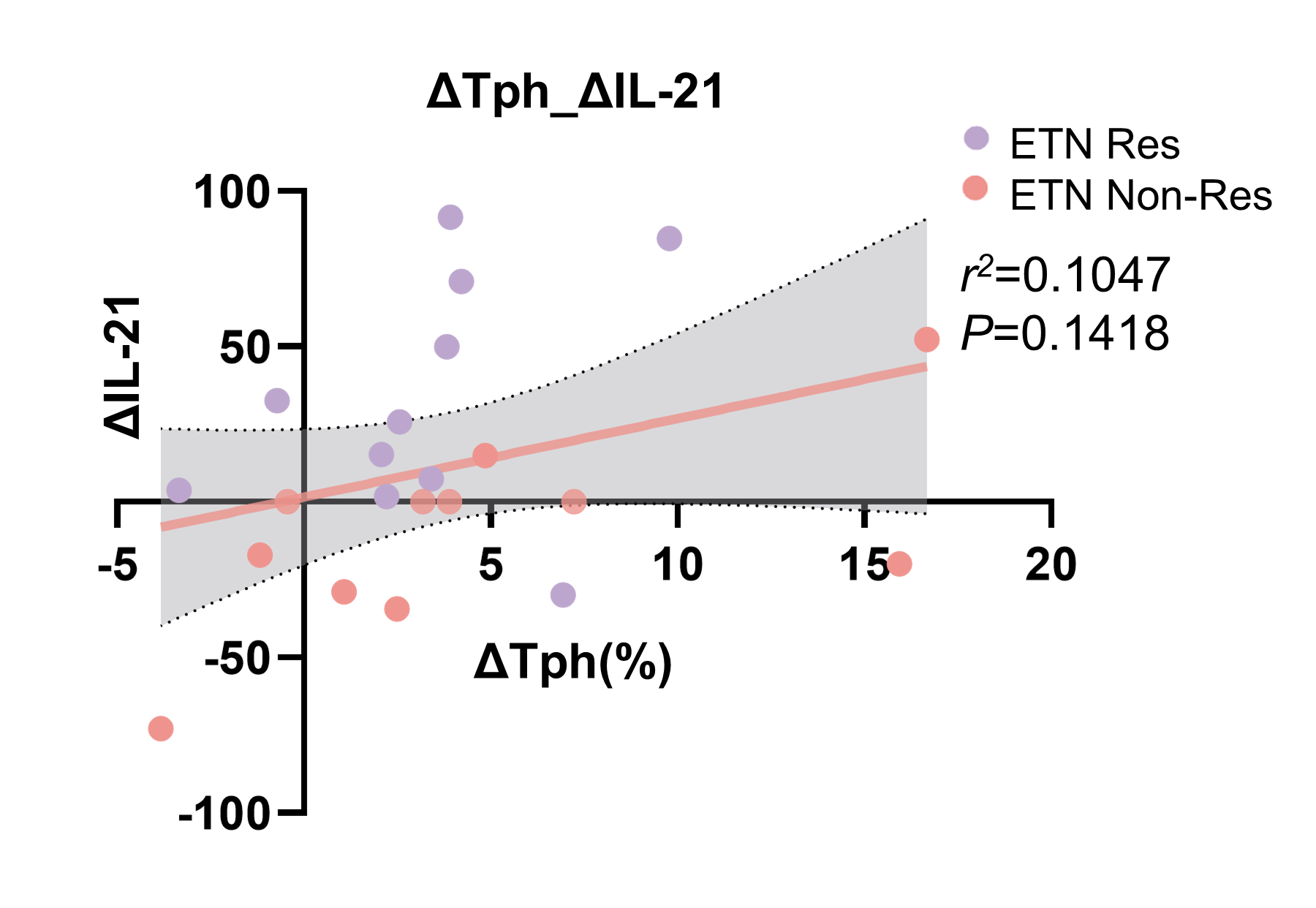


**Figure S7. Gating strategy:**

(1) Tph cell (PD‑1^hi^CXCR5⁻CD4⁺ T cell)


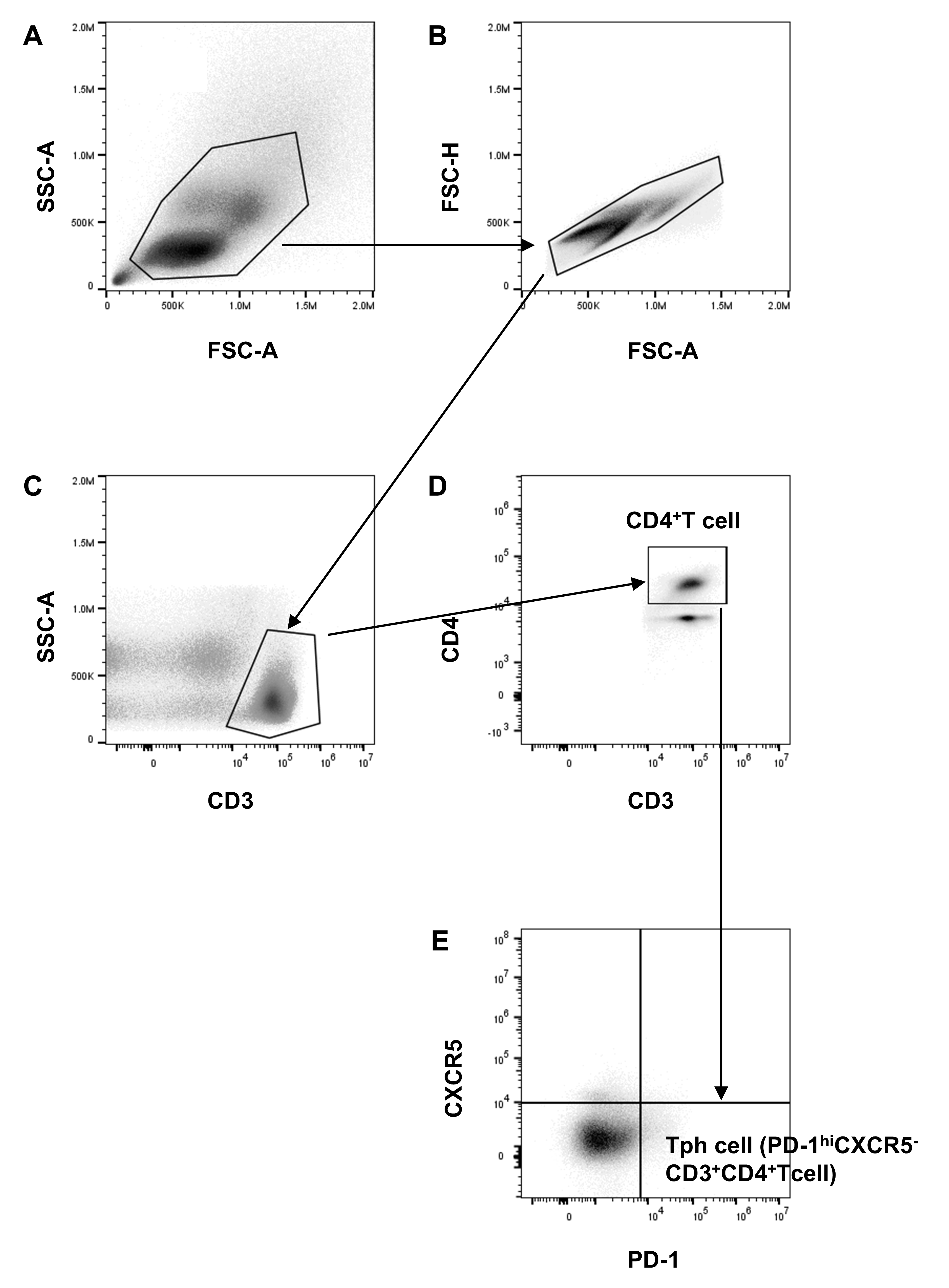


(2) Tfh cell (PD‑1^hi^CCR7^+^CXCR5^+^CD4⁺ T cell); Naïve Th cell (CD3^+^CD4^+^CD45RA^+^)


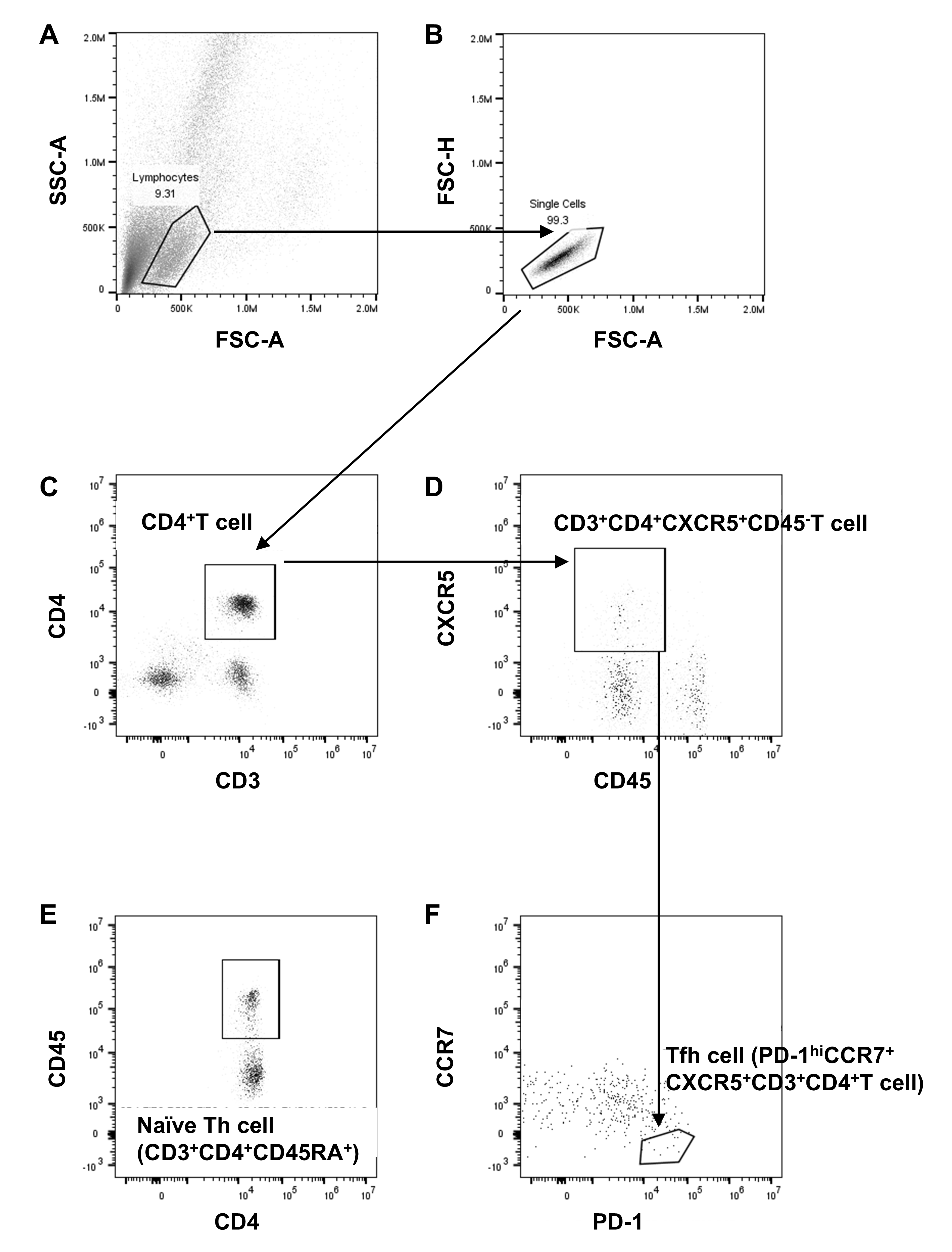


(3) Foxp3^+^Treg cell (CD3^+^CD4^+^CD25^hi^Foxp3^+^); Teff cell (CD3^+^CD4^+^CD25^low^Foxp3^-^)


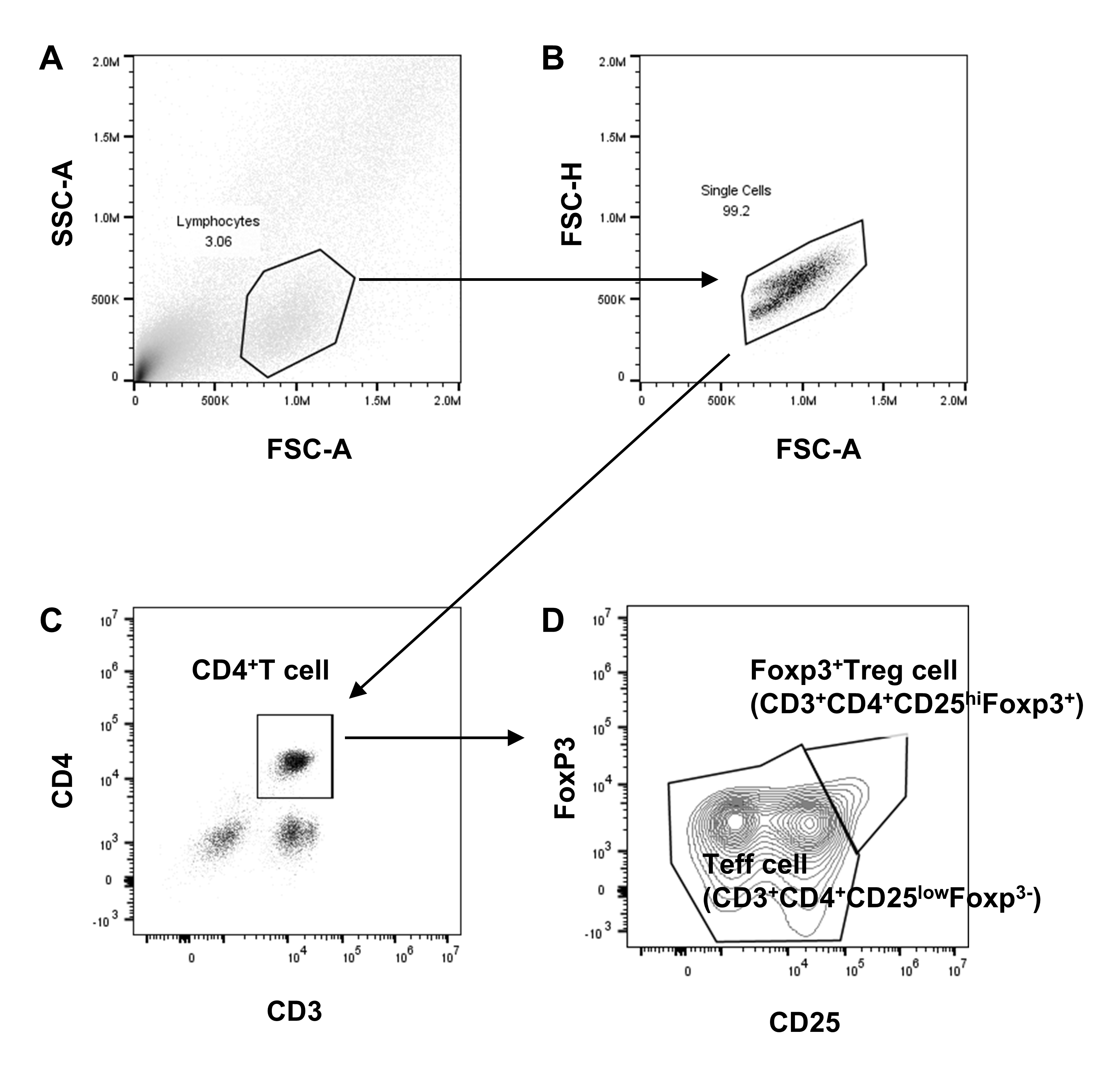


(4) CLA^+^Treg cell (CLA^+^CD25^hi^CD127^low^T cell); CD161^+^Treg cell (CD161^+^ CD25^hi^CD127^low^T cell)


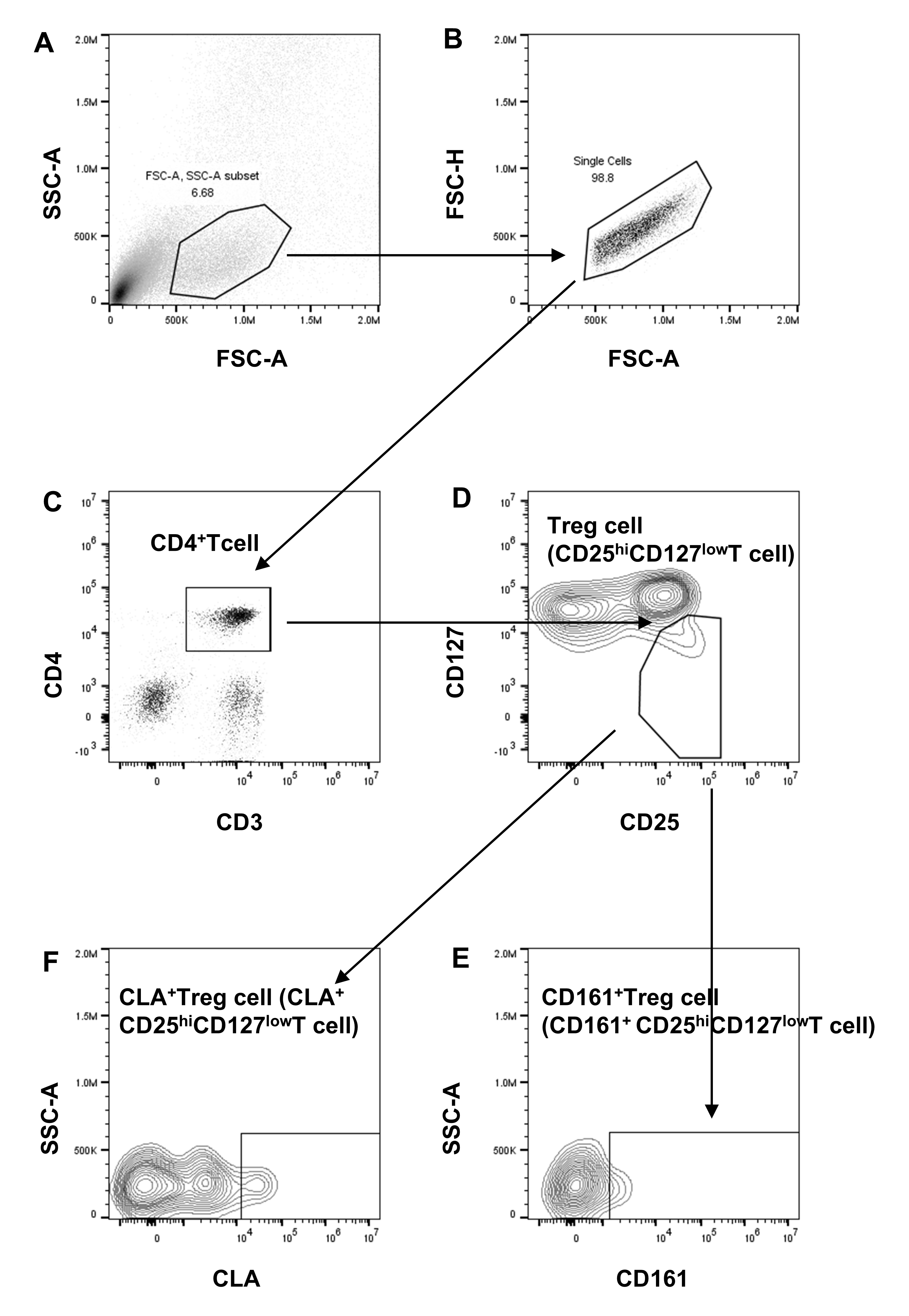


(5) TNF-α^+^Th1 cell; IFN-γ^+^Th1 cell; IL-2^+^Th1 cell; Th17 cell


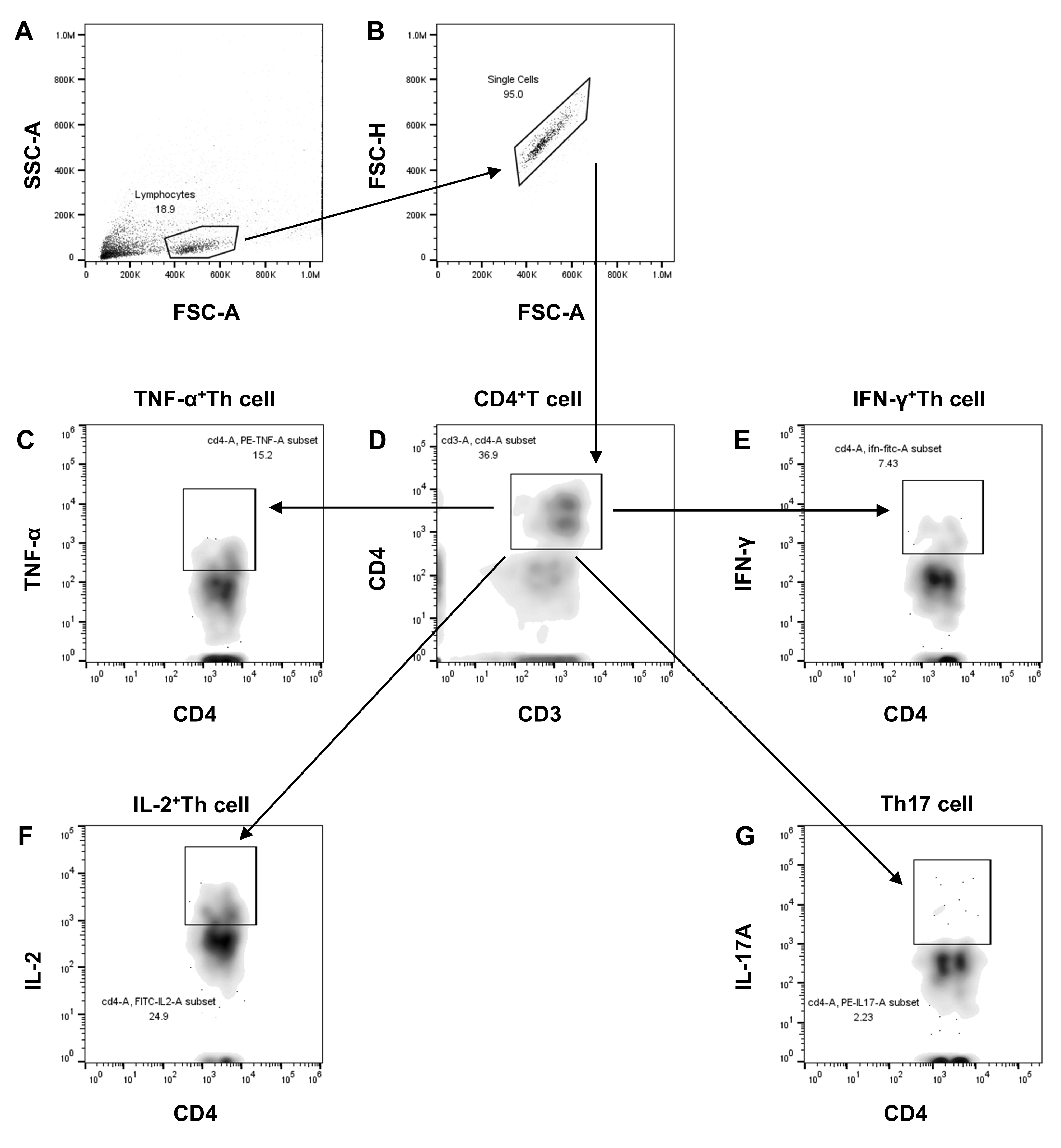

Supplement: Supplementary file 1 — Supplementary File1: Table S1: Demographic and clinical characteristics of RA patients. Table S2: Baseline demographic characteristics of study groups. Table S3: (A) Univariate assessment of the association between immune cell subsets and DAS28‐CRP scores. (B) Multivariable assessment of the association between immune cell subsets and DAS28‐CRP scores. Table S4: Markers and representation of immune cell subsets. Table S5: Flow cytometry fluorescent antibodies. Figure S1: Subgroup analysis of Tph cell frequency stratified by csDMARDs regimens. Figure S2: The percentage of Tph cells was positively correlated with (A) WBC and (B) PLT, while negatively correlated with (C) HGB. Figure S3: Correlation analysis of Tph cell frequency with clinical and laboratory parameters. (A) age, (B) duration, (C) Sex, (D) smoking, (E) alcohol, (F) alanine transaminase (ALT), (G) blood urea nitrogen (BUN), (H) creatinine (Cr), (I) rheumatoid factor (RF), (J) anti‐cyclic citrullinated peptide antibody (Anti‐CCP Ab). Figure S4: Correlations between Tph cells and additional composite disease activity indices. (A) Correlation between Tph cell frequency and continuous CDAI. (B) Correlation between Tph cell frequency and continuous SDAI. (C) Progressive increase in Tph cell levels across four‐category CDAI classification. (D) Progressive increase in Tph cell levels across four‐category SDAI classification. Figure S5: Receiver operating characteristic curve and area under the curve (AUC) value of Tph cells. Figure S6: Correlation between changes in serum IL‑21 levels (ΔIL‑21) and changes in circulating Tph cell frequency (ΔTph) in RA patients following etanercept treatment. Figure S7: Gating strategy: (1) Tph cell (PD‑1hiCXCR5−CD4+ T cell). (2) Tfh cell (PD‑1hiCCR7+CXCR5+CD4+ T cell); Naïve Th cell (CD3+CD4+CD45RA+). (3) Foxp3+ Treg cell (CD3+CD4+CD25hiFoxp3+); Teff cell (CD3+CD4+CD25lowFoxp3−). (4) CLA+Treg cell (CLA+CD25hiCD127lowT cell); CD161+Treg cell (CD161+ CD25hiCD127lowT cell). (5) TNF [file MCO2-7-e70876-s001.docx]
